# Dopamine Metabolism as a Chemically Constrained Network: Structural Regulation, Redox Liability and Dopaminergic Vulnerability in Parkinson’s Disease

**DOI:** 10.3390/medsci14060608

**Published:** 2026-09-27

**Authors:** Emmanuel Ortega-Robles, Magdalena Guerra-Crespo, Oscar Arias-Carrión

**Affiliations:** 1 División de Neurociencias, Clínica, Instituto Nacional de Rehabilitación Luis Guillermo Ibarra Ibarra, Mexico City 14389, Mexico; edortegar@gmail.com; 2 Laboratory of Regenerative Medicine, Department of Physiology, Faculty of Medicine, National Autonomous University of Mexico, Mexico City 04510, Mexico; mguerra@facmed.unam.mx; 3 Escuela de Medicina y Ciencias de la Salud, Tecnologico de Monterrey, Mexico City 14380, Mexico

**Keywords:** dopamine metabolism, redox chemistry, tyrosine hydroxylase, monoamine oxidase, neuromelanin, Parkinson’s disease

## Abstract

Dopamine metabolism lies at the intersection of enzymatic catalysis, redox chemistry and cellular compartmentalisation, forming a tightly regulated network that sustains neurotransmission while limiting intrinsic chemical reactivity. In catecholaminergic neurons, dopamine is both an essential signalling molecule and a redox-active substrate prone to oxidation, aldehyde formation and quinone chemistry, requiring coordinated synthesis, storage, degradation and detoxification. Recent advances in structural biology and chemical enzymology have revealed detailed conformational regulation and specific protein interactions among key components of this system—including tyrosine hydroxylase, aromatic L-amino acid decarboxylase, monoamine oxidase and catechol-O-methyltransferase—while suggesting that their spatial organisation may contribute to the control of metabolic flux and exposure to reactive intermediates. Building on previous work on dopamine dyshomeostasis, oxidative stress and reactive metabolites, this Review addresses a less explored integrative question: how structural organisation, catalytic chemistry, intracellular compartmentalisation and redox control collectively influence neuronal resilience. Dopamine metabolism is therefore conceptualised as a chemically constrained and structurally integrated network in which cofactor dynamics, vesicular sequestration, enzyme interactions and redox buffering regulate metabolic flux while limiting reactive intermediates. Disruption of this network—through impaired compartmentalisation, cofactor imbalance, oxidative and nitrosative stress, or neuroinflammatory signalling—can shift dopamine toward electrophilic and oxidative toxicity, contributing to nigrostriatal vulnerability. Neuromelanin formation, reactive nitrogen species signalling and aldehyde accumulation further link dopamine metabolism to mitochondrial dysfunction and immune activation. This framework highlights limitations of therapies centred predominantly on dopamine replacement and supports mechanism-driven approaches aimed at restoring metabolic and redox homeostasis in Parkinson’s disease.

## 1. Introduction

Dopamine occupies a singular position at the interface of neurotransmission, metabolism, and redox chemistry, where its physiological necessity is inseparable from its intrinsic chemical liability [1,2]. Within catecholaminergic neurons, dopamine is not merely a signalling molecule but also a reactive metabolite whose synthesis, transformation, and compartmentalisation must be tightly orchestrated to preserve cellular integrity [3]. This duality—function and hazard—defines the biological paradox of dopaminergic systems and underpins the selective vulnerability of nigrostriatal neurons in Parkinson’s disease (PD) [4,5].

From a molecular standpoint, dopamine metabolism constitutes a structurally coordinated, chemically constrained network rather than a linear biochemical pathway [6]. Enzymatic catalysis, cofactor availability, vesicular transport, and redox buffering operate in concert to maintain a dynamic equilibrium between neurotransmitter production and oxidative containment [6]. Tyrosine hydroxylase, aromatic L-amino acid decarboxylase (AADC), and their associated cofactors form the core biosynthetic machinery [7,8]. Nevertheless, their activity is inseparable from the spatial organisation of intracellular compartments, the thermodynamics of proton gradients, and the kinetics of electron transfer reactions [3]. This integrated system reflects an evolutionary solution to a fundamental problem: how to produce and deploy a highly reactive catecholamine without incurring cumulative molecular damage.

The chemical nature of dopamine imposes stringent constraints on neuronal physiology. The catechol moiety is inherently susceptible to oxidation, generating semiquinones, quinones, and reactive oxygen species that can covalently modify proteins, disrupt membranes, and impair mitochondrial function [9,10]. Consequently, dopaminergic neurons rely on rapid vesicular sequestration, efficient enzymatic degradation, and robust detoxification pathways to limit cytosolic exposure to reactive intermediates [6]. Failure of any component within this network—whether through impaired enzyme regulation, disrupted vesicular transport, or altered cofactor metabolism—shifts dopamine from a signalling molecule to a source of oxidative and electrophilic stress [11].

Over the past decade, advances in structural biology, cryo-electron microscopy, and chemical enzymology have transformed our understanding of dopamine metabolism [8,12]. These approaches have revealed that key enzymes operate through coordinated conformational states, transient high-energy intermediates, and specific protein–protein interactions, challenging simplified textbook representations [7,13]. Such findings raise the possibility that spatial organisation may contribute to the coordination of dopamine metabolism and to limiting exposure to reactive intermediates. However, higher-order biosynthetic assemblies and substrate channelling between dopamine-metabolising enzymes have not been directly demonstrated in dopaminergic neurons and should therefore be regarded as mechanistic hypotheses rather than established features of the pathway [14]. This emerging framework positions dopamine metabolism as a self-organising biochemical system, whose stability depends on both structural integrity and chemical control.

In parallel, there is growing recognition that dopamine metabolism is deeply embedded within broader cellular networks, including mitochondrial bioenergetics, metal homeostasis, and neuroimmune signalling [9,15,16]. Oxidative and nitrosative stress, protein aggregation, and neuroinflammation are not secondary consequences but integral components of dopaminergic dysfunction [10,16]. Within this context, Parkinson’s disease emerges not simply as a disorder of dopamine deficiency, but as a systems-level failure of neurometabolic homeostasis, in which perturbations in synthesis, degradation, and redox balance converge to drive progressive neurodegeneration [17].

Despite this expanding mechanistic insight, an important conceptual gap remains between different levels of dopamine biology. Previous reviews have extensively addressed the complexity of dopamine metabolism, dopamine dyshomeostasis and oxidative stress in PD, as well as specific pathogenic mechanisms such as DOPAL accumulation and the catecholaldehyde hypothesis [6,11,18]. These studies have established the importance of cytosolic dopamine, reactive metabolites, impaired vesicular sequestration, and defective detoxification in dopaminergic vulnerability. However, these processes have less frequently been integrated with emerging structural and mechanistic information on the enzymes and transporters that govern dopamine flux, including their conformational regulation, cofactor dependence, macromolecular interactions, and spatial organisation. Consequently, the relationship between molecular architecture, intracellular compartmentalisation, chemical reactivity, and network-level metabolic failure remains incompletely synthesised.

This narrative Review addresses this gap by examining dopamine metabolism as a chemically constrained and structurally coordinated network, rather than primarily as a linear pathway or as a collection of individual toxic mechanisms. We integrate structural biology and chemical enzymology with evidence concerning cofactor dynamics, vesicular sequestration, dopamine degradation and detoxification, redox buffering, and neuron–glia interactions. Figure 1 summarises this framework, illustrating how these processes cooperate to restrict the intracellular exposure of dopamine and its reactive intermediates and how disruption at multiple nodes can shift the system toward oxidative and electrophilic stress. By connecting molecular-scale mechanisms with cellular vulnerability and therapeutic strategies, this Review aims to provide a framework for understanding how failure of dopamine handling may contribute to neurometabolic dysfunction in PD and for identifying intervention points beyond symptomatic dopamine replacement.

## 2. Dopamine Biosynthesis

Dopamine biosynthesis is not simply a neurotransmitter-producing pathway; it is a chemically constrained and stringently buffered metabolic system whose dysregulation may contribute to the vulnerability of catecholaminergic neurons [6]. Figure 2 provides an integrated overview of this biosynthetic architecture, illustrating the sequential coordination of tetrahydrobiopterin (BH_4_) production, tyrosine hydroxylation, L-DOPA decarboxylation, vesicular sequestration, and synaptic release, together with a conceptual model of possible spatial coordination among these processes.

In the central nervous system, dopamine biosynthesis must support activity-dependent neurotransmission while restricting the cytosolic accumulation of a chemically reactive catecholamine. The pathway comprises TH-mediated conversion of L-tyrosine to L-DOPA, AADC-mediated decarboxylation to dopamine, and rapid VMAT2-dependent vesicular sequestration. These sequential steps depend on distinct cofactors and regulatory mechanisms and are summarised in Figure 2 and Table 1. The following subsections examine these components separately: Section 2.1 and Section Regulation of Tyrosine Hydroxylase address the structural basis and regulation of TH catalysis, Section 2.2 and Section GTPCH–TH Interaction examine BH_4_ metabolism and the evidence for GTPCH–TH interaction, Section 2.3 considers AADC structure and function, and Section 2.4 addresses VMAT2-dependent vesicular sequestration.

### 2.1. Tyrosine Hydroxylase

Tyrosine hydroxylase (TH) is the kinetic and chemical gatekeeper of catecholamine biosynthesis, catalysing the rate-limiting hydroxylation of L-tyrosine to L-DOPA [7]. Beyond its canonical placement in the pathway, TH represents a redox-active metalloenzyme whose catalytic cycle, conformational plasticity, and cofactor dependence position it at the intersection of neurotransmission and oxidative metabolism. As a member of the aromatic amino acid hydroxylase family, TH shares a conserved catalytic logic with phenylalanine and tryptophan hydroxylases, yet its physiological context—within long-lived, highly active dopaminergic neurons—renders its chemistry uniquely consequential for cellular vulnerability [7,17].

Structurally, TH assembles as a homotetramer (~240 kDa). Each protomer contains an intrinsically disordered N-terminal regulatory region, a catalytic domain harbouring a non-haem Fe(II) centre, and a C-terminal oligomerisation domain that stabilises quaternary structure [7,12]. The catalytic iron is coordinated by a conserved 2-His-1-carboxylate motif and functions together with molecular oxygen and tetrahydrobiopterin (BH_4_). Catalysis proceeds through oxygen activation and formation of highly reactive iron–oxygen intermediates, including a ferryl Fe(IV)=O species that mediates aromatic hydroxylation of L-tyrosine [7]. BH_4_ participates directly in this chemistry as an electron donor; its biosynthesis, recycling, and redox regulation are considered separately in Section 2.2.

Human TH is expressed as four splice isoforms (TH1–TH4) that share the same catalytic core but differ within their N-terminal regulatory regions, producing differences in regulatory behaviour [7]. Recent structural studies have provided important insight into how this N-terminal region controls access to catalytically competent conformations [12]. These structural features provide the molecular basis for feedback inhibition, phosphorylation-dependent activation, and proteostatic regulation, which are discussed in the following section.

#### Regulation of Tyrosine Hydroxylase

TH activity is controlled through complementary mechanisms that allow dopamine synthesis to respond rapidly to neuronal demand while limiting excessive catecholamine production [23,24,25]. A major regulatory mechanism is catecholamine-mediated feedback inhibition. Dopamine binding stabilises an autoinhibitory configuration involving the N-terminal regulatory region and the catalytic domain, reducing access to catalytically competent states [7,12].

Phosphorylation of the N-terminal regulatory domain provides rapid and reversible control of TH activity. Ser40 phosphorylation is particularly important because it weakens dopamine-mediated inhibition and promotes catalytic activation [12,24]. Ser19 and Ser31 phosphorylation have distinct regulatory effects; Ser19 facilitates interaction with 14-3-3 proteins, whereas Ser31 can modulate catalytic activity and cofactor-related properties [24,25,26]. Protein phosphatases, including PP2A and PP2C, counterbalance these kinase-dependent mechanisms and return TH toward its basal phosphorylation state [24].

TH regulation is also coupled to protein stability and cellular redox conditions. Oxidative and nitrosative modifications can impair catalytic activity, while ubiquitin–proteasome-dependent degradation removes damaged or misfolded enzyme [25,27]. Chaperone systems provide an additional regulatory layer. In particular, DNAJC12 interacts directly with TH and contributes to maintenance of its structural integrity [13], whereas 14-3-3 proteins stabilise specific phosphorylated states [25,26]. Other reported protein interactions, including those involving DJ-1 and α-synuclein, may further influence TH activity under physiological or pathological conditions [28,29]. The evidence and physiological relevance of direct interaction between TH and GTPCH are considered separately in Section GTPCH–TH Interaction.

Oxygen availability also influences TH catalysis because molecular oxygen is an obligate substrate. Experimental studies indicate that changes in oxygen concentration can alter TH activity and the behaviour of its Fe-dependent catalytic cycle [30]. This factor is particularly relevant when interpreting biochemical experiments performed under oxygen conditions that differ from those encountered in brain tissue.

Much of the mechanistic understanding of TH regulation derives from biochemical and non-human experimental systems [24,30]. Although recent structural studies have substantially advanced knowledge of human TH, the integration of phosphorylation, protein interactions, redox state, and catalytic regulation in human dopaminergic neurons remains incompletely defined. Human iPSC-derived dopaminergic neurons and related models therefore provide an important platform for determining how these regulatory mechanisms operate in a physiologically relevant cellular context [31].

### 2.2. BH_4_ Metabolism and GTP Cyclohydrolase I

Tetrahydrobiopterin is a redox-active cofactor directly involved in the catalytic chemistry of aromatic amino acid hydroxylases. In TH, BH_4_ participates in molecular oxygen activation and electron transfer required for hydroxylation of L-tyrosine. It is therefore better considered a co-substrate in a coupled redox reaction than a passive enzymatic accessory [7,32].

During TH-mediated hydroxylation, BH_4_ is oxidised to a 4a-hydroxytetrahydropterin intermediate, which subsequently forms quinonoid dihydrobiopterin (qBH_2_). Regeneration of BH_4_ requires pterin-4a-carbinolamine dehydratase (PCD) and dihydropteridine reductase (DHPR), allowing the cofactor to remain in its reduced, catalytically competent state [32]. Efficient recycling is important not only for sustaining catalytic flux but also for limiting accumulation of oxidised pterins that may favour enzyme uncoupling and reactive oxygen species formation.

De novo BH_4_ synthesis begins with guanosine triphosphate (GTP), with GTP cyclohydrolase I (GTPCH, encoded by GCH1) catalysing the rate-limiting conversion of GTP to dihydroneopterin triphosphate. GTPCH forms a homodecameric toroidal complex in which active sites are located at subunit interfaces. This oligomeric architecture supports coordinated catalysis and regulation of BH_4_ production in response to cellular demand [32].

BH_4_ homeostasis depends on the balance between synthesis, recycling, and oxidation. Insufficient BH_4_ limits TH catalytic turnover and dopamine synthesis, whereas supraphysiological BH_4_ concentrations can undergo auto-oxidation and promote redox stress. Experimental studies in dopaminergic cells have also associated elevated BH_4_ with mitochondrial dysfunction and differential inhibition of respiratory-chain complexes [33]. Thus, both cofactor deficiency and excessive accumulation can perturb cellular homeostasis [6,19].

GTPCH is regulated at several levels. cAMP-dependent signalling can enhance GCH1 expression, whereas GTPCH activity is modulated through interaction with GTP cyclohydrolase I feedback regulatory protein (GFRP). BH_4_ promotes GFRP-dependent feedback inhibition, while phenylalanine acts as a positive effector. GTPCH phosphorylation, particularly at Ser81, can further increase activity and reduce susceptibility to GFRP-dependent inhibition [19]. These mechanisms allow BH_4_ production to respond dynamically to metabolic and signalling demands.

BH_4_ also serves as an obligate cofactor for nitric oxide synthases, linking pterin metabolism to cellular redox and nitric oxide signalling. Although relevant to broader BH_4_ biology, these functions extend beyond its direct role in dopamine biosynthesis [19].

The clinical importance of this pathway is illustrated by GCH1 mutations causing dopa-responsive dystonia, in which impaired BH_4_ synthesis limits dopamine production and produces a marked response to levodopa therapy [19,34]. Alterations in BH_4_ metabolism have also been implicated in PD, where they may intersect with impaired TH activity, mitochondrial dysfunction, and oxidative stress [19,35]. However, the extent to which BH_4_ dysregulation contributes directly to PD pathogenesis remains incompletely established.

#### GTPCH–TH Interaction

The functional relationship between GTP cyclohydrolase I (GTPCH) and TH is firmly established at the metabolic level, because GTPCH controls the rate-limiting step in the synthesis of BH_4_, which is required for TH-mediated hydroxylation of L-tyrosine. Evidence also indicates that this relationship may extend beyond cofactor provision through direct protein–protein interaction. However, the strength and physiological relevance of this evidence depend strongly on the experimental system and should be interpreted accordingly [36,37].

The most detailed evidence for direct GTPCH–TH interaction derives from studies using *Drosophila* proteins and experimental models. Bowling et al. demonstrated physical association between TH and GTPCH using co-immunoprecipitation from *Drosophila* head extracts, recombinant-protein pull-down and far-Western assays, and yeast two-hybrid experiments [36]. These experiments also identified interaction regions within the proteins and showed that phosphorylation can influence the association in an isoform-dependent manner. Importantly, the same study demonstrated functional effects of the interaction under defined biochemical conditions: GTPCH increased TH activity, including an increase in apparent maximal velocity without a corresponding change in substrate affinity, while TH increased GTPCH activity and reduced its susceptibility to BH_4_-mediated feedback inhibition [36]. These findings provide evidence for mutual functional regulation in this experimental system, but they do not establish a structurally defined allosteric mechanism.

Evidence that such an interaction can also occur between the human proteins has subsequently emerged. Human wild-type TH and GTPCH were co-immunoprecipitated following expression in mammalian cell lines, and some disease-associated TH variants reduced this interaction [37]. This observation supports conservation of the capacity for TH–GTPCH association beyond *Drosophila*. Nevertheless, these experiments were performed in heterologous cellular systems rather than endogenous human dopaminergic neurons. Thus, the stoichiometry, dynamics, regulatory significance, and contribution of the interaction to physiological dopamine synthesis in human neurons remain unresolved.

Phosphorylation provides an additional level of regulation for both enzymes, although its relationship to TH–GTPCH association should also be distinguished from the regulation of each enzyme individually. TH phosphorylation, particularly at Ser40, promotes catalytic activation by relieving dopamine-mediated inhibition [12], whereas GTPCH phosphorylation at Ser81 can enhance enzyme activity and reduce susceptibility to GFRP-dependent inhibition [38]. The latter mechanism has been demonstrated experimentally in non-neuronal cellular systems [38]. While the *Drosophila* studies indicate that phosphorylation can modulate TH–GTPCH association under specific conditions [36], current evidence does not establish a coordinated phosphorylation-dependent feedforward mechanism between human TH and GTPCH in dopaminergic neurons.

The available kinetic and genetic observations are also compatible with functional cooperation beyond simple changes in bulk BH_4_ concentration. For example, reductions in GTPCH activity in *Drosophila* were associated with reduced TH activity that could not be fully restored by exogenous BH_4_, suggesting that additional regulatory mechanisms may contribute to their functional relationship [36]. However, these findings do not demonstrate direct transfer of BH_4_ between the enzymes, localised cofactor delivery, or metabolic channelling. Likewise, there is currently insufficient evidence to establish that TH and GTPCH form a discrete catalytic microdomain in dopaminergic neurons. These possibilities remain mechanistically plausible hypotheses requiring direct spatial and functional validation.

Importantly, direct GTPCH–TH interaction does not replace the established regulatory mechanisms governing either enzyme. Dopamine-mediated feedback regulation of TH and GFRP-dependent regulation of GTPCH remain major determinants of their respective activities [36,39]. The interaction observed experimentally may therefore represent an additional regulatory layer superimposed on these established mechanisms rather than a distinct, obligatory regulatory module.

The potential clinical relevance of this interaction should also be interpreted cautiously. Alterations in GCH1 expression and BH_4_ metabolism are relevant to disorders of dopamine synthesis, including dopa-responsive dystonia, and genetic variation in GCH1 has also been associated with PD [34,40]. However, these observations do not demonstrate that disruption of the physical TH–GTPCH interaction contributes to PD pathogenesis. Similarly, although patient-derived TH variants can affect TH–GTPCH association in heterologous cells [37], the contribution of this mechanism to disease phenotypes remains to be established in physiologically relevant neuronal models.

Accordingly, the TH–GTPCH interaction represents an intriguing additional level of regulation within dopamine biosynthesis, but its physiological role remains incompletely defined. Future studies using endogenous proteins in mammalian dopaminergic neurons, particularly human iPSC-derived or other physiologically relevant neuronal models, will be necessary to determine whether TH–GTPCH association regulates local BH_4_ availability or dopamine synthesis under physiological conditions and whether this interaction is altered in PD. Until such evidence becomes available, localised BH_4_ delivery, metabolic channelling, and therapeutic targeting of the TH–GTPCH interaction should be regarded as testable hypotheses rather than established mechanisms.

### 2.3. Aromatic L-Amino Acid Decarboxylase

Aromatic L-amino acid decarboxylase (AADC; DDC) catalyses the conversion of L-DOPA to dopamine and therefore occupies a central position in monoamine biosynthesis. Although traditionally regarded as a downstream, non-rate-limiting enzyme, structural and biochemical evidence indicates that its activity can influence dopaminergic output and respond dynamically to changes in substrate and neurotransmitter availability [6]. AADC also participates in the synthesis of serotonin and trace amines, extending its functional relevance beyond the dopaminergic system.

At the molecular level, AADC is a pyridoxal 5′-phosphate (PLP)-dependent homodimer, with active sites formed at the dimer interface. PLP is covalently linked to an active-site lysine and acts as an electron sink during decarboxylation. Catalysis proceeds through formation of an external aldimine with L-DOPA, followed by decarboxylation, generation of a quinonoid intermediate, protonation, and release of dopamine. Recent structural studies indicate that human AADC exhibits asymmetric conformational states and catalytic-loop dynamics, suggesting that intersubunit communication contributes to enzymatic turnover and substrate processing [8].

AADC is regulated at several levels. Alternative promoter usage and splicing contribute to tissue-specific expression [41,42], whereas enzymatic activity is influenced by PLP availability, phosphorylation, and receptor-mediated signalling [6,43]. Dopaminergic signalling can also modulate AADC activity: dopamine receptor antagonism has been associated with increased activity, whereas receptor activation can suppress it [6]. These observations indicate that AADC behaves as a regulated component of dopamine synthesis rather than as a constitutively active downstream enzyme.

Metabolic and pharmacological perturbations further illustrate this regulation. MAO inhibition, which increases intracellular dopamine availability, has been associated with reduced AADC activity, whereas conditions of dopamine depletion can produce compensatory increases [6]. These responses are context-dependent and vary across experimental models, cell types, and species, underscoring the need for caution when extrapolating individual regulatory mechanisms to human dopaminergic neurons.

Because AADC generates dopamine directly in the cytosol, its activity is functionally linked to subsequent vesicular sequestration. Insufficient VMAT2-dependent storage increases the fraction of cytosolic dopamine available for enzymatic and non-enzymatic oxidation [10,14]. The structural basis and protective role of VMAT2-dependent dopamine storage are discussed in Section 2.4, while the consequences of altered dopamine partitioning for degradation and oxidative metabolism are considered in Section 3 and Section 5.1, respectively.

The clinical relevance of AADC is particularly evident during levodopa therapy in PD. L-DOPA crosses the blood–brain barrier through large neutral amino acid transporters and is subsequently converted to dopamine by AADC in surviving dopaminergic neurons and, particularly as nigrostriatal denervation advances, in non-dopaminergic cells including serotonergic terminals [6,44]. Dopamine production and release from these alternative cellular sources can become less tightly regulated and has been implicated in treatment-related motor complications, including dyskinesias [39,44,45]. AADC has also been targeted experimentally through viral vector-mediated gene delivery to the striatum, with clinical studies demonstrating increased local AADC activity and potential symptomatic benefit in advanced PD [46,47]. These therapeutic approaches are discussed further in Section 7.

Despite these advances, important questions remain regarding AADC regulation in vivo. The extent to which conformational dynamics, PLP availability, phosphorylation, and protein interactions determine enzymatic behaviour in human dopaminergic neurons remains incompletely understood [8]. Clarifying these mechanisms will be important for defining how AADC contributes to dopamine homeostasis under physiological conditions and during chronic dopaminergic therapy.

### 2.4. Vesicular Sequestration by VMAT2

Newly synthesised dopamine is rapidly transported from the cytosol into synaptic vesicles by vesicular monoamine transporter 2 (VMAT2; SLC18A2), a process essential both for neurotransmitter storage and for limiting the chemical reactivity of cytosolic dopamine. Recent cryo-EM structures of human VMAT2 have clarified the molecular basis of this process, revealing conformational states associated with substrate and inhibitor binding and supporting a proton-coupled transport mechanism driven by the vesicular electrochemical gradient [48]. The acidic vesicular lumen favours dopamine retention and reduces its exposure to cytosolic oxidative chemistry.

Vesicular sequestration also has an important protective function. Reduced VMAT2 activity increases the fraction of dopamine remaining in the cytosol, thereby favouring spontaneous oxidation and MAO-mediated conversion to DOPAL. Experimental models with impaired vesicular storage show increased vulnerability of nigrostriatal dopaminergic neurons, whereas enhanced VMAT2 activity can be neuroprotective [6]. Post-mortem neurochemical studies have also suggested reduced vesicular uptake in PD, although the extent to which this represents a primary pathogenic mechanism rather than a consequence of neurodegeneration remains uncertain.

VMAT2 therefore provides the principal interface between dopamine biosynthesis and intracellular compartmentalisation. Its relationship with MAO-mediated metabolism and DOPAL formation is considered further in Section 3.

## 3. Dopamine Degradation

Dopamine degradation comprises interconnected enzymatic pathways whose relative contribution depends on cellular localisation and substrate availability. Cytosolic dopamine can undergo oxidative deamination by monoamine oxidases (MAO-A and MAO-B), generating 3,4-dihydroxyphenylacetaldehyde (DOPAL), which is subsequently detoxified predominantly by aldehyde dehydrogenases (ALDHs) to 3,4-dihydroxyphenylacetic acid (DOPAC). DOPAC can then be O-methylated by catechol-O-methyltransferase (COMT) to form homovanillic acid (HVA), while additional conjugation pathways contribute to metabolite elimination [6,20].

These reactions differ importantly in their chemical and cellular consequences: MAO generates a reactive aldehyde intermediate, ALDH limits its accumulation, and COMT and conjugation pathways contribute to downstream inactivation and clearance. Figure 3 summarises these routes and their relationship to competing oxidative reactions. The following subsections examine the structural and catalytic basis of MAO, ALDH-dependent DOPAL detoxification, COMT-mediated methylation, and phase II metabolism, with particular attention to the strength and limitations of evidence linking alterations in these pathways to PD.

### 3.1. Monoamine Oxidase (MAO)

Monoamine oxidase is a flavin-dependent oxidoreductase that initiates a major pathway of intracellular monoamine degradation. By catalysing the oxidative deamination of dopamine and other monoamines, MAO regulates neurotransmitter turnover while generating aldehyde products whose subsequent detoxification is required to maintain cellular homeostasis [6,10].

MAO exists as two isoforms, MAO-A and MAO-B, encoded by distinct genes but sharing a conserved overall architecture. Both enzymes are anchored to the outer mitochondrial membrane through a C-terminal transmembrane segment, with their catalytic domains exposed to the cytosol. Each contains a covalently bound flavin adenine dinucleotide (FAD) cofactor that mediates substrate oxidation. Although structurally related, MAO-A and MAO-B differ in active-site geometry and gating residues, resulting in distinct substrate preferences and inhibitor sensitivities [49].

Catalysis involves oxidation of the monoamine substrate with concomitant reduction of FAD, generating an iminium intermediate that is subsequently hydrolysed to the corresponding aldehyde. Reoxidation of reduced FAD by molecular oxygen completes the catalytic cycle. For dopamine, this reaction generates DOPAL, which must be rapidly detoxified by downstream aldehyde-metabolising pathways [6,20]. The chemistry and pathogenic significance of DOPAL are considered separately in Section 3.2.

The contribution of MAO-A and MAO-B to dopamine metabolism requires careful distinction between experimental systems and cellular compartments. MAO-A preferentially metabolises serotonin and norepinephrine but also contributes substantially to intraneuronal dopamine deamination. Experimental studies, particularly in rodent catecholaminergic systems, support a prominent role for MAO-A in the metabolism of cytosolic dopamine, whereas MAO-B is highly abundant in the brain and is strongly expressed in astrocytes and other non-neuronal populations [50]. Consequently, the relative contribution of each isoform varies with species, brain region, cell type, age, and pharmacological conditions, and findings obtained with one system should not be assumed to apply directly to human nigrostriatal neurons.

The distinction is illustrated by MPTP metabolism. In the brain, MPTP is converted predominantly by MAO-B, particularly in astrocytic compartments, to the active metabolite MPP^+^ [51]. MPP^+^ is then taken up by dopaminergic neurons through the dopamine transporter (DAT) and accumulates in mitochondria, where it inhibits complex I and impairs oxidative phosphorylation [15,52]. This pathway underlies the widespread use of MPTP as an experimental model of parkinsonism, but the prominent role of MAO-B in MPTP bioactivation should not be taken as evidence that MAO-B is likewise the principal enzyme responsible for endogenous intraneuronal dopamine deamination.

The redox consequences of MAO activity also warrant qualification. In the canonical biochemical reaction, reoxidation of FAD by molecular oxygen generates H_2_O_2_, providing a potential link between increased MAO flux and oxidative stress [6,9]. However, extrapolation from isolated enzymatic reactions to intact catecholaminergic cells may be overly simplistic. Recent cellular studies indicate that MAO-dependent dopamine metabolism can engage a mitochondrial electron-transfer shuttle that stimulates the respiratory chain, rather than necessarily producing a freely diffusible cytosolic H_2_O_2_ burden [53]. Accordingly, the contribution of MAO-derived H_2_O_2_ to oxidative stress is likely to depend on subcellular context, mitochondrial function, and antioxidant capacity. The broader chemistry of dopamine-derived ROS and Fenton reactions is discussed in Section 5.1.

MAO-mediated dopamine turnover is also determined by the amount of dopamine accessible in the cytosol. Vesicular sequestration limits substrate availability for oxidative deamination, whereas impaired storage can shift dopamine toward MAO-dependent metabolism [6,20]. Because VMAT2-dependent compartmentalisation is addressed specifically in Section 2.4, its mechanism is not repeated here.

Overall, MAO represents an important interface between dopamine turnover and mitochondrial metabolism, but its contribution to dopaminergic vulnerability cannot be reduced to a simple relationship between increased enzyme activity and oxidative stress. Isoform-specific expression, cell type, substrate availability, mitochondrial coupling, and downstream DOPAL detoxification all influence the biological consequences of MAO activity [6,10,17,20]. MAO-B inhibition remains clinically important for symptomatic treatment of PD, while its broader metabolic and potential disease-modifying implications are considered in Section 7 [39].

### 3.2. Aldehyde Detoxification and ALDH

A critical determinant of the chemical fate of cytosolic dopamine is the detoxification of DOPAL, the reactive catecholaldehyde generated by MAO-mediated oxidative deamination. Under physiological conditions, DOPAL is predominantly oxidised by ALDHs to the less reactive DOPAC in an NAD^+^-dependent reaction. A secondary pathway mediated by aldehyde/aldose reductases converts DOPAL to DOPET, but this route contributes substantially less to DOPAL clearance under normal conditions [11,18,20]. Consistent with this predominance, the DOPAC:DOPAL ratio in control human putamen greatly exceeds the DOPET:DOPAL ratio, whereas impaired ALDH activity shifts metabolism toward DOPET without fully compensating for reduced oxidative detoxification [20]. Thus, ALDH activity constitutes a major biochemical barrier limiting intracellular exposure to DOPAL.

Within dopaminergic neurons, DOPAL detoxification is attributed principally to cytosolic ALDH1A1 and mitochondrial ALDH2 [11]. These enzymes share approximately 70% sequence identity and closely related tetrameric architectures, although ALDH2 contains an N-terminal mitochondrial targeting sequence. ALDH1A1 contains catalytic, cofactor-binding, and oligomerisation domains, including a Rossmann-fold NAD(P)^+^-binding region and a conserved catalytic cysteine required for aldehyde oxidation. High-resolution structures of human ALDH1A1 demonstrate substantial similarity to ALDH2, particularly within their active and cofactor-binding regions, which also presents a challenge for isoform-selective pharmacological modulation [54]. Their cellular distribution nevertheless differs: ALDH1A1 is expressed by specific populations of midbrain dopaminergic neurons, with ALDH1A1-positive cells concentrated predominantly in the ventral tier of the human substantia nigra pars compacta (SNpc), whereas ALDH2 has broader tissue distribution and resides within mitochondria [11,21]. Reported K_m_ values of approximately 0.4 μM for ALDH1A1 and 1 μM for ALDH2 are consistent with efficient DOPAL metabolism at low intracellular concentrations [11].

The biological importance of this pathway arises from the unusual chemical reactivity of DOPAL. Its aldehyde group can modify primary amines, particularly lysine residues, whereas oxidation of its catechol moiety generates reactive semiquinone and quinone species capable of modifying thiols such as cysteine [11]. Experimental studies consequently associate DOPAL with protein cross-linking, enzyme inhibition, oxidative stress, and impaired proteostasis. α-Synuclein is a particularly relevant target: DOPAL promotes its oligomerisation and formation of high-molecular-weight species in biochemical and cellular systems [11,18,20,21]. DOPAL can also impair mitochondrial function and promote mitochondrial permeability transition and energetic failure [11,18]. These observations establish the biochemical plausibility of DOPAL toxicity, but their contribution to human PD must be distinguished from effects demonstrated under experimental conditions.

Genetic models provide additional support while also illustrating the limitations of a simple ALDH-deficiency model. Loss of ALDH1A1 can enhance DOPAL-associated and proteinase K-resistant α-synuclein species in α-synuclein transgenic models, whereas ALDH1A1 overexpression can protect cultured midbrain dopaminergic neurons against α-synuclein-mediated toxicity [21]. However, ALDH1A1 deficiency alone does not reproduce a robust PD-like phenotype, consistent with partial functional compensation by other aldehyde-detoxifying enzymes. More pronounced abnormalities occur when both Aldh1a1 and Aldh2 are disrupted, producing age-dependent DOPAL accumulation, motor impairment, and nigrostriatal degeneration [11,20]. Even these models do not reproduce the full neurochemical pattern or magnitude of dopaminergic loss observed in human PD, indicating that impaired aldehyde detoxification is more likely to interact with other pathogenic processes than to act as an isolated determinant.

Human evidence is suggestive but requires particularly careful interpretation. In post-mortem putamen from patients with advanced PD, absolute DOPAL concentrations were lower than in controls, consistent with the profound depletion of dopaminergic tissue; however, DOPAL relative to residual dopamine was increased approximately fivefold [20]. The DOPAC:DOPAL ratio was also reduced by approximately 70%, from which a comparable reduction in effective ALDH activity was inferred using a kinetic model [20]. Importantly, this represents an indirect estimate based on tissue metabolite ratios, rather than a direct measurement of ALDH activity in living dopaminergic neurons. Reduced ALDH1A1 gene and protein expression has also been reported in PD substantia nigra, and ALDH1A1-positive ventral SNpc populations are markedly depleted in post-mortem PD tissue [11,21]. However, reduced ALDH1A1 expression may be either a contributor to vulnerability or, at least in part, a consequence of preferential loss of ALDH1A1-expressing neurons. Thus, the directionality of this association remains unresolved.

ALDH activity may also be influenced by processes associated with oxidative stress. Lipid-peroxidation products such as 4-hydroxynonenal and malondialdehyde can inhibit aldehyde detoxification, while sufficiently elevated DOPAL can inhibit its own ALDH-mediated metabolism [11,18]. These observations raise the possibility of positive-feedback interactions between oxidative stress and aldehyde accumulation, although their quantitative importance in human dopaminergic neurons remains uncertain. Environmental toxicants provide another experimental route to ALDH impairment. Benomyl and its metabolites inhibit ALDH, alter dopamine metabolism, and selectively damage dopaminergic neurons in cellular and animal models [55]. Reduction in DOPAL formation by MAO inhibition mitigated toxicity in these experiments, particularly that induced by the ALDH-inhibiting benomyl metabolite MBT, supporting DOPAL accumulation as one contributing mechanism. Higher estimated occupational exposure to benomyl was also associated with increased PD risk in an epidemiological analysis [55]. However, these observational data cannot establish causality or demonstrate that ALDH inhibition constitutes a general pathogenic mechanism in sporadic PD.

Collectively, these biochemical, experimental, and human observations support the catecholaldehyde hypothesis, in which insufficient detoxification increases exposure to DOPAL and may promote interactions with α-synuclein, mitochondrial dysfunction, oxidative stress, and impaired dopamine handling [18]. However, they do not establish a causal role for ALDH dysfunction or DOPAL accumulation in human PD. The evidence is strongest for the chemical reactivity of DOPAL and for the consequences of experimentally manipulating ALDH activity; evidence that impaired ALDH-mediated detoxification is a primary driver of human PD remains more indirect. Accordingly, the catecholaldehyde hypothesis is best regarded as a mechanistically supported component of a multifactorial model of dopaminergic vulnerability rather than an established unitary explanation for PD.

From a translational perspective, the DOPAC:DOPAL ratio and related catechol profiles provide useful mechanistic indices in experimental and post-mortem studies [20], but their application as clinical biomarkers remains limited because DOPAL is reactive, present at low concentrations, and not reliably detected in human cerebrospinal fluid [11,20]. Likewise, enhancement of aldehyde detoxification remains an interesting therapeutic concept, but the structural similarity and broad physiological functions of ALDH isoenzymes complicate selective pharmacological targeting [54]. These limitations emphasise the need for approaches capable of measuring DOPAL, its macromolecular adducts, and ALDH function directly in physiologically relevant human neuronal systems.

### 3.3. Catechol-O-Methyltransferase (COMT)

Catechol-O-methyltransferase catalyses the O-methylation of catechol substrates using SAM as the methyl donor and Mg^2+^ as an essential catalytic cofactor. Within dopamine metabolism, COMT complements MAO-dependent oxidative deamination by methylating dopamine and its downstream metabolites. In the major degradative pathway, COMT converts DOPAC to HVA, an abundant terminal metabolite of dopamine metabolism [6].

Structurally, COMT adopts a compact α/β fold in which Mg^2+^ coordinates the catechol substrate and facilitates methyl transfer from SAM. The reaction proceeds through direct SN2 displacement, yielding an O-methylated product and S-adenosylhomocysteine. Substrate orientation within the active site determines regioselectivity, and catalytic properties vary according to substrate and enzyme isoform [56].

Two principal COMT isoforms arise through alternative promoter usage: soluble COMT (S-COMT) and membrane-bound COMT (MB-COMT), which contains an additional N-terminal membrane-anchoring sequence. The two isoforms share the same catalytic core but differ in subcellular localisation and kinetic properties. MB-COMT has higher affinity for catechol substrates and is therefore considered particularly relevant at the relatively low catechol concentrations encountered in the brain, whereas S-COMT displays greater catalytic capacity at higher substrate concentrations and contributes substantially to the metabolism of circulating and exogenous catechols [22]. These differences illustrate that the contribution of COMT depends strongly on both substrate availability and cellular context.

This context dependence is particularly evident within the central nervous system. COMT expression is heterogeneous and occurs predominantly in non-dopaminergic and glial compartments, with comparatively low expression in nigrostriatal dopaminergic neurons [57]. Its contribution to dopamine clearance therefore varies across brain regions. In the striatum, where DAT-mediated reuptake is highly efficient, COMT has a relatively limited role in terminating extracellular dopamine signalling. By contrast, in regions such as the prefrontal cortex, where DAT expression is sparse, COMT contributes substantially to dopamine clearance and the regulation of extracellular dopamine tone [58]. Thus, COMT should not be regarded as a uniformly dominant route of dopamine inactivation throughout the brain.

O-methylation also modifies the chemical properties of catechol substrates. Masking one hydroxyl group reduces catechol character and can decrease susceptibility to oxidation and redox cycling, although the quantitative importance of COMT to neuronal redox protection relative to vesicular sequestration and MAO-dependent metabolism remains uncertain [6]. Accordingly, COMT-mediated methylation is better considered a pathway toward less redox-reactive and more readily eliminated metabolites, rather than a conversion of catechols into completely “metabolically inert” species.

The clinical relevance of COMT is well established through pharmacological inhibition. COMT inhibitors such as entacapone and opicapone reduce peripheral L-DOPA metabolism and prolong its availability during PD treatment [39,56,59]. Their principal clinical effect is therefore pharmacokinetic rather than evidence that altered endogenous COMT activity is itself a major driver of PD pathogenesis; their therapeutic use is discussed further in Section 7. Genetic variation also influences COMT activity. The common Val158Met polymorphism affects enzyme thermal stability and catalytic activity and has been associated particularly with differences in cortical dopamine signalling and related cognitive phenotypes [22,60]. However, its relevance to nigrostriatal dopamine degradation or PD pathogenesis is less direct.

Overall, COMT provides a compartment- and region-dependent route for catechol methylation whose physiological importance varies with substrate concentration, cellular localisation, and the availability of alternative clearance mechanisms. This contextual role distinguishes COMT from the predominantly intraneuronal MAO–ALDH pathway and emphasises that the relative contribution of individual degradative routes cannot be inferred independently of brain region and cellular environment [6,58].

### 3.4. Phase II Conjugation and Complementary Quinone Detoxification

Phase II conjugation provides an additional route for the metabolism and elimination of dopamine and other catechols through sulphation and glucuronidation. These reactions increase metabolite polarity and generally reduce the accessibility of the catechol hydroxyl groups to oxidation, thereby favouring clearance toward less redox-reactive products. However, their quantitative contribution differs markedly between peripheral tissues and the central nervous system, and they appear to play a secondary role relative to MAO-, ALDH-, and COMT-dependent pathways in brain dopamine turnover [6].

Sulphation is catalysed by cytosolic sulfotransferases (SULTs), particularly SULT1A3, using 3′-phosphoadenosine-5′-phosphosulphate (PAPS) as the sulphate donor. Structural studies of human SULT1A3 have defined the molecular determinants of catechol substrate recognition [61], while biochemical analyses demonstrate preferential formation of dopamine 3-O-sulphate, providing a mechanistic explanation for its predominance over the corresponding 4-O-sulphate in human circulation [62]. Thus, the regioselectivity of dopamine sulphation is supported primarily by structural and in vitro enzymatic evidence and is consistent with the distribution of circulating conjugates.

Glucuronidation is mediated by endoplasmic-reticulum-associated UDP-glucuronosyltransferases (UGTs), which transfer glucuronic acid from UDP-glucuronic acid (UDPGA) to catechol substrates. Human UGT1A10 can glucuronidate dopamine and shows lower positional selectivity than SULT1A3, allowing formation of both 3-O- and 4-O-glucuronides [62]. However, UGT1A10 is primarily expressed in peripheral tissues, and the enzymes responsible for dopamine glucuronidation within the human brain remain incompletely characterised [63,64].

This distinction is important because phase II metabolism is substantially more prominent in the periphery than in the brain. Sulphate and glucuronide conjugates contribute appreciably to systemic catechol clearance, whereas unconjugated DOPAC and HVA remain major dopamine metabolites in the central nervous system [6]. Nevertheless, intact sulphate and glucuronide conjugates of dopamine-related metabolites have been detected in human brain microdialysates and cerebrospinal fluid, demonstrating that these reactions also occur centrally [64]. Their abundance, enzymatic origin, regional distribution, and quantitative contribution to dopamine turnover in human dopaminergic neurons remain poorly defined.

Conjugated catechols may also have analytical value as indicators of dopamine metabolism. Metabolomic approaches can resolve alterations across multiple dopamine-derived products [65], and a recent analysis of the Parkinson’s Progression Markers Initiative cohort identified cerebrospinal-fluid dopamine 3-O-sulphate as a potential predictor of motor complications in PD [66]. However, such associations should be distinguished from evidence that altered phase II conjugation contributes directly to PD pathogenesis. At present, the evidence is stronger for a role of sulphation and glucuronidation in catechol clearance than for their involvement as primary determinants of nigrostriatal degeneration.

Beyond sulphation and glucuronidation, dopamine-derived quinones are controlled by complementary enzymatic detoxification mechanisms. In particular, NAD(P)H:quinone oxidoreductase 1 (NQO1; DT-diaphorase) catalyses the two-electron reduction of aminochrome to leukoaminochrome, thereby limiting formation of the redox-cycling leukoaminochrome o-semiquinone radical. Glutathione transferase M2-2 (GSTM2) provides an additional protective pathway by catalysing glutathione conjugation of dopamine o-quinone and aminochrome, reducing their electrophilic reactivity and propensity to participate in redox cycling [67,68]. Experimental studies further indicate that astrocyte-derived GSTM2 can be transferred to dopaminergic cells, suggesting a potential intercellular component of quinone detoxification. These mechanisms are particularly relevant because aminochrome has been associated experimentally with mitochondrial dysfunction, oxidative stress, impairment of proteasomal and autophagy–lysosomal pathways, endoplasmic reticulum stress, α-synuclein oligomerisation, and neuroinflammatory responses.

Overall, sulphation and glucuronidation represent context-dependent terminal clearance mechanisms, with well-established roles in peripheral catechol metabolism but less clearly defined contributions within the human brain. These pathways complement other protective mechanisms, including NQO1- and GSTM2-dependent detoxification of dopamine-derived quinones (Table 2). Further identification of the relevant CNS SULT and UGT isoforms, together with quantitative assessment of quinone-detoxifying pathways in human dopaminergic systems, will be required to establish their relative contribution to dopamine homeostasis and their potential relevance to PD [64,67,68].

## 4. Context-Dependent Differences in Dopamine Metabolism

Dopamine metabolism is not a uniform biochemical process but a context-dependent network whose behaviour varies across species, tissues, and cellular microenvironments. These differences arise from divergent enzyme expression, subcellular organisation, cofactor availability, and physicochemical constraints, and collectively shape both the kinetics and the chemical consequences of dopamine turnover. As such, variability in dopaminergic metabolism is not a confounding artefact but a defining property of the system, with direct implications for mechanistic interpretation and translational inference [6].

A major source of complexity lies in species-specific differences in enzymatic architecture and substrate handling. For example, the relative contribution of monoamine oxidase isoforms differs markedly between humans and rodents, with both MAO-A and MAO-B contributing to dopamine deamination in the human brain in a region-dependent manner, whereas MAO-A appears to account for most physiological striatal dopamine degradation in rodents [50,69]. Similarly, the expression and functional relevance of enzymes such as COMT, ALDH, and vesicular transporters vary across species, altering both the flux distribution through metabolic pathways and the profile of intermediate metabolites. These differences extend beyond enzyme abundance to include kinetic parameters, cofactor interactions, and regulatory mechanisms, complicating direct extrapolation from preclinical models to human neurobiology [6,50,69,70].

At the organ level, dopamine metabolism is partitioned between central and peripheral compartments, each governed by distinct biochemical rules. Remarkably, a substantial proportion of total body dopamine is synthesised outside the brain, particularly within the gastrointestinal tract, where it functions in paracrine and endocrine signalling rather than classical neurotransmission. This peripheral dopamine pool is subject to different enzymatic constraints, oxygen tensions, and metabolic fluxes, resulting in metabolite profiles that diverge from those observed in the central nervous system. Consequently, systemic measurements of dopamine or its metabolites represent a composite signal integrating multiple tissues, rather than a direct readout of neuronal activity [71].

This distinction becomes particularly problematic when relying on peripheral biomarkers, such as urinary or plasma levels of HVA or DOPAC, to infer central dopaminergic function. While analytically accessible, these measures lack spatial specificity and are influenced by hepatic metabolism, renal clearance, and peripheral enzymatic activity. From a chemical standpoint, they reflect the end products of multiple converging pathways, obscuring the contribution of individual processes such as vesicular sequestration, aldehyde detoxification, or local redox dynamics. As a result, peripheral biomarkers may fail to capture the compartmentalised and transient nature of neuronal dopamine metabolism, limiting their interpretative value in both research and clinical settings [72].

At the cellular level, dopamine metabolism is further shaped by cell-type-specific specialisation and intercellular coupling. Neurons, astrocytes, and microglia express distinct complements of metabolic enzymes and transporters, resulting in a division of biochemical labour. For instance, dopaminergic neurons prioritise synthesis and vesicular storage, whereas glial cells disproportionately contribute to extracellular metabolism via enzymes such as COMT and MAO-B [50,57]. This arrangement creates a distributed metabolic system in which dopamine clearance and detoxification depend on coordinated interactions between cell types. Moreover, subcellular compartmentalisation—spanning cytosol, synaptic vesicles, mitochondria, and endoplasmic reticulum—introduces additional layers of regulation, as each compartment imposes distinct pH, redox potential, and substrate accessibility constraints [6].

Recent technological advances have begun to resolve this complexity with unprecedented resolution. Positron emission tomography (PET) imaging using radiotracers targeting dopamine synthesis, transport, and receptor binding enables region-specific quantification of dopaminergic function in vivo [73]. Complementary approaches in metabolomics now allow detailed profiling of dopamine-derived metabolites across tissues, capturing broad metabolic signatures and pathway alterations [65]. At the cellular scale, single-cell transcriptomics and spatial omics have revealed previously unrecognised heterogeneity in enzyme expression, cofactor metabolism, and stress-response pathways within dopaminergic circuits [74]. Together, these tools are transforming dopamine metabolism from a bulk measurement into a multidimensional, compartment-resolved system.

Human-derived experimental models further bridge the translational gap. Induced pluripotent stem cell (iPSC)-derived dopaminergic neurons recapitulate key aspects of human-specific metabolism, including enzyme expression patterns and susceptibility to oxidative stress [10]. When combined with genome editing and patient-specific genetic backgrounds, these models provide a platform for interrogating how genetic variation, environmental exposures, and metabolic context converge on dopaminergic dysfunction [31].

From a translational perspective, these observations necessitate a shift away from reductionist, tissue-agnostic models toward a framework that explicitly incorporates biological context. Dopamine should not be treated as a homogeneous entity, but as a metabolite whose behaviour depends on where, how, and under what conditions it is produced and degraded [6]. This has direct implications for biomarker development: future strategies must prioritise compartment-specific indicators that distinguish central from peripheral processes and capture early perturbations in neuronal metabolism [75].

Clinically, embracing this complexity may enable more precise patient stratification and targeted therapeutic interventions. For example, distinguishing between defects in synthesis, vesicular storage, degradation, or detoxification could inform personalised treatment strategies, rather than relying on uniform dopamine replacement. Integration of imaging, metabolomic, and genetic data may further allow dynamic monitoring of disease progression and treatment response [65,75].

In summary, metabolic heterogeneity is a fundamental feature of dopamine biology, arising from the interplay of species, tissue, cellular, and subcellular determinants [6]. Recognising this complexity transforms dopamine metabolism from a linear pathway into a context-dependent network and highlights the need for integrative approaches that bridge molecular mechanisms with systems-level physiology. Such a perspective is essential for advancing both mechanistic understanding and clinical management of disorders such as Parkinson’s disease.

## 5. Catecholamines, Oxidative Stress and Inflammation

### 5.1. Dopamine Oxidation and Oxidative Stress

Dopamine metabolism is intrinsically coupled to redox chemistry, positioning catecholamines at the centre of neuronal vulnerability. Beyond its function as a neurotransmitter, dopamine is a chemically labile catechol whose oxidation generates a spectrum of reactive intermediates that can perturb protein function, mitochondrial integrity, and cellular signalling. This dual identity—signalling molecule and redox-active substrate—renders dopamine metabolism a biochemical liability that must be tightly constrained to preserve neuronal homeostasis [10].

A principal source of oxidative stress arises from MAO-mediated deamination, which produces H_2_O_2_ alongside aldehyde intermediates. However, this enzymatic pathway represents only one component of a broader oxidative landscape. Dopamine and its precursor L-DOPA are inherently prone to autoxidation, particularly under conditions of elevated cytosolic concentration, alkaline pH, or increased transition metal availability. In the presence of redox-active iron, dopamine undergoes one-electron oxidation to form semiquinones and superoxide radicals, which rapidly evolve into dopamine quinones—highly electrophilic species capable of widespread molecular damage [10,17,76]. Dopamine oxidation, metal-dependent redox cycling, quinone formation, and their relevance to nigral vulnerability are extensively treated in the dopamine-oxidation literature; however, although this chemistry provides a mechanistic basis for L-DOPA-associated oxidative effects observed in experimental systems, it does not establish that therapeutic levodopa increases neurodegeneration in patients with PD.

These quinone intermediates occupy a central position in dopamine-derived toxicity. Chemically, they undergo intramolecular cyclisation reactions to form aminochrome and related indole derivatives, intermediates in the biosynthesis of neuromelanin. While neuromelanin may function as a long-term sink for redox-active species, its formation reflects a cumulative burden of catechol oxidation [9,76].

Among the downstream products of dopamine oxidation, aminochrome has received particular attention because of its reactivity and experimentally demonstrated effects on several cellular systems. In cellular, organotypic, and animal models, aminochrome has been associated with mitochondrial dysfunction, impaired autophagy–lysosomal and proteasomal function, endoplasmic reticulum stress, cytoskeletal disruption, α-synuclein oligomerisation, oxidative stress, and glial activation [77,78,79,80]. More recent evidence further indicates that aminochrome can disrupt autophagosome–lysosome fusion, providing an additional mechanism linking dopamine oxidation to impaired proteostasis. These observations support aminochrome as an important experimentally characterised dopamine-derived reactive intermediate, although its quantitative contribution to neurodegeneration in human PD remains to be established.

Under pathological conditions, dopamine oxidation may also generate neurotoxic derivatives such as 6-hydroxydopamine, while dopamine can independently give rise to tetrahydroisoquinolines such as salsolinol, which impair mitochondrial respiration, inhibit key metabolic enzymes, and propagate oxidative stress [81].

At the molecular level, dopamine oxidation products exert their effects by covalently modifying proteins. Quinones react preferentially with nucleophilic residues, particularly cysteine thiols, forming adducts that alter protein conformation, disrupt catalytic activity, and impair intracellular trafficking. Proteins central to dopaminergic function—including the DAT and TH—are especially susceptible to such modifications, creating a direct mechanistic link between oxidative chemistry and dysregulated neurotransmission [6,76].

A critical target of dopamine-derived oxidative stress is α-synuclein, a protein whose aggregation defines the pathology of Parkinson’s disease. Dopamine and its oxidation products stabilise soluble protofibrillar forms of α-synuclein, inhibiting its normal physiological interactions while promoting the formation of aggregation-prone species. These assemblies can adopt membrane-disruptive conformations, compromise vesicular integrity, and amplify cytosolic dopamine exposure, thereby establishing a self-reinforcing cycle of oxidative stress and protein aggregation [29].

From a chemical perspective, dopamine oxidation involves a network of competing reactions that are governed by substrate concentration, metal availability, pH, and antioxidant capacity. The balance between enzymatic degradation, vesicular sequestration, and non-enzymatic oxidation determines whether dopamine is safely metabolised or diverted into toxic pathways. Disruption of this balance—through impaired vesicular storage, mitochondrial dysfunction, or altered metal homeostasis—shifts dopamine metabolism toward pro-oxidant states, particularly in the substantia nigra, where iron levels are elevated and antioxidant defences are relatively limited [9].

Cells deploy multiple mechanisms to mitigate these risks. O-methylation by COMT reduces the redox reactivity of catechols by masking hydroxyl groups, thereby limiting quinone formation and metal-catalysed oxidation [6]. In parallel, conjugation with glutathione provides a major detoxification route for dopamine quinones, forming stable adducts that can be further processed and eliminated [82]. However, excessive oxidative burden can deplete glutathione reserves, impairing this protective system and exacerbating cellular vulnerability [82].

Importantly, oxidative dopamine metabolism is tightly linked to neuroinflammatory signalling. Reactive oxygen species and quinone-modified proteins can activate microglia, induce pro-inflammatory cytokine release, and disrupt neuronal–glial homeostasis. This interplay between oxidative stress and inflammation establishes a feed-forward loop, in which metabolic dysfunction amplifies immune activation, further increasing oxidative damage and neuronal loss [83].

These insights fundamentally challenge the traditional view of dopamine as an unequivocally beneficial neurotransmitter. Instead, dopamine emerges as a molecule with inherent biochemical risk, whose physiological utility depends on precise control of its synthesis, compartmentalisation, and degradation [76]. Ageing, genetic susceptibility, and environmental exposures can all destabilise this balance, tipping the system toward oxidative injury and neurodegeneration [17].

From a translational standpoint, this reframing has profound implications. Therapeutic strategies should extend beyond dopamine replacement to include stabilisation of dopamine metabolism, modulation of redox-active metal ions, enhancement of antioxidant defences, and optimisation of intracellular compartmentalisation. Targeting the chemistry of dopamine itself—rather than solely its signalling function—may prove essential for developing disease-modifying interventions [9,76].

In summary, dopamine oxidation is not an incidental by-product of neurotransmission but a central biochemical process that shapes neuronal fate. The principal reactive intermediates generated during dopamine metabolism, together with their chemical properties, molecular targets, and downstream consequences, are summarised in Table 3. Their integration with redox chemistry, protein modification, and inflammatory signalling defines a core axis of neurodegenerative vulnerability [83]. A mechanistic understanding of this axis, grounded in structural and chemical principles, is essential for advancing both fundamental neuroscience and therapeutic innovation.

### 5.2. Neuromelanin

Neuromelanin (NM) is a redox-active, polymeric pigment that accumulates selectively within catecholaminergic neurons of the substantia nigra and locus coeruleus—cell populations that exhibit disproportionate vulnerability in Parkinson’s disease. Far from being an inert by-product, NM represents a chemically complex endpoint of dopamine oxidation, integrating quinone chemistry, metal coordination, and macromolecular assembly into a long-lived intracellular structure. Its composition includes oxidised catecholamine-derived oligomers, covalently modified proteins, lipids, and tightly bound transition metals, organised through a combination of covalent linkages and non-covalent interactions such as π–π stacking and metal chelation [6,9].

At the chemical level, NM formation originates from dopamine quinone intermediates generated during cytosolic oxidation. These electrophilic species undergo a cascade of reactions, including intramolecular cyclisation to aminochrome, subsequent polymerisation, and covalent conjugation with nucleophilic residues of proteins and thiol-containing molecules such as cysteine and glutathione. The resulting structure resembles a heterogeneous, eumelanin-like polymer, but with a distinct composition reflecting its catecholaminergic origin. Iron and other transition metals are incorporated during this process, stabilising the polymer matrix while simultaneously conferring redox activity [9,76].

A key determinant of NM formation is the intracellular distribution of dopamine. Experimental evidence indicates that NM is derived primarily from cytosolic, non-vesicular dopamine, rather than from dopamine safely compartmentalised within synaptic vesicles. Consistent with this, enhanced expression of VMAT2—which promotes vesicular sequestration—reduces NM accumulation, underscoring the principle that NM formation reflects failure of compartmentalisation and excess cytosolic dopamine exposure [84]. Once formed, NM is sequestered within autophagic–lysosomal organelles, where it accumulates over decades, suggesting that it represents a long-term cellular strategy for isolating chemically reactive species [84,85].

Functionally, NM exhibits a dual, context-dependent role. Under physiological conditions, it acts as a protective sink, sequestering dopamine-derived quinones and limiting their interaction with cellular macromolecules. Its high affinity for transition metals—particularly iron—further enhances this protective capacity by reducing the availability of redox-active metal ions that would otherwise participate in Fenton chemistry, generating highly reactive hydroxyl radicals. In this capacity, NM helps buffer the oxidative burden associated with catecholamine metabolism [9].

However, this protective function is inherently finite. With ageing and sustained metabolic activity, NM progressively accumulates iron and other redox-active species, increasing its potential to act as a catalytic centre for oxidative reactions. When a critical threshold is exceeded, the NM–metal complex may transition from a redox buffer to a redox amplifier, promoting oxidative stress rather than suppressing it [9]. Moreover, oxidative damage or lysosomal dysfunction can destabilise NM-containing organelles, leading to the release of bound metals and quinone-derived intermediates into the cytosol, thereby amplifying cellular injury [85].

A further pathological dimension emerges upon neuronal death. Extracellular release of NM into the surrounding parenchyma triggers microglial activation and sustained neuroinflammatory responses, reflecting its recognition as a damage-associated molecular pattern. This process establishes a feed-forward cycle in which oxidative stress promotes neuronal loss, releasing NM, which in turn drives inflammation and further degeneration. This interplay between dopamine metabolism, NM accumulation, and immune activation represents a central axis in the pathogenesis of Parkinson’s disease [86,87].

From a structural perspective, NM is remarkably resistant to enzymatic degradation, and no defined catabolic pathway has been identified. Its persistence suggests that NM accumulation is effectively irreversible over the lifespan, reinforcing its role as a long-term repository for reactive metabolites. However, this same property raises the possibility of cumulative toxicity, as progressive accumulation increases the burden of bound metals and oxidised species within vulnerable neurons [85].

Recent technological advances have enabled in vivo interrogation of NM biology. Neuromelanin-sensitive magnetic resonance imaging exploits the paramagnetic properties of NM–metal complexes to visualise NM-containing neurons in the human brain. This approach provides a non-invasive biomarker of dopaminergic neuron integrity, with potential applications in early diagnosis, disease staging, and longitudinal monitoring of Parkinson’s disease [88,89].

These observations support a dual role for NM, which can sequester redox-active metals and dopamine-derived products intracellularly but may also contribute to inflammatory signalling when released from degenerating neurons [9,84]. However, the extent to which manipulating NM formation, metal loading, or clearance can alter the course of PD remains uncertain. Approaches aimed at modifying iron homeostasis, dopamine oxidation, or lysosomal handling are mechanistically relevant, but their effects on NM biology have been defined predominantly in experimental systems and have not established NM modulation as a disease-modifying strategy in patients [9,85].

Neuromelanin should therefore be regarded primarily as an informative interface between dopamine metabolism, metal handling, and cumulative cellular stress. Neuromelanin-sensitive MRI provides a promising means of assessing the integrity of pigmented nigral neurons [88,89], whereas therapeutic manipulation of NM dynamics remains an experimental concept requiring further validation.

### 5.3. Oxidative Stress, Inflammation and Neurodegeneration

Oxidative stress and neuroinflammation constitute closely interacting processes that may contribute to dopaminergic vulnerability in PD, rather than acting solely as downstream consequences of degeneration. Within dopaminergic systems, this axis is intrinsically embedded in metabolism: dopamine synthesis, vesicular leakage, and enzymatic degradation collectively generate ROS, electrophilic aldehydes, and quinone intermediates, establishing a basal redox load that must be continuously buffered. The failure of this buffering capacity transforms physiological turnover into a self-amplifying cascade of oxidative injury [6,10,83].

At the chemical level, oxidative stress reflects an imbalance between ROS production and detoxification. Dopamine metabolism contributes directly to this imbalance through MAO-mediated hydrogen peroxide generation, autoxidation of cytosolic catechols, and iron-catalysed redox cycling [6,9]. Under physiological conditions, antioxidant systems—including superoxide dismutase, catalase, and glutathione-dependent peroxidases—maintain redox equilibrium. However, in the substantia nigra, these defences are comparatively limited, and glutathione depletion has been consistently observed, reducing the capacity to neutralise ROS and detoxify quinone species [90]. This creates a biochemical environment in which even modest increases in oxidative flux can have disproportionate cellular consequences.

The downstream effects of ROS are structurally and functionally pervasive. Lipid peroxidation compromises membrane integrity and generates secondary reactive aldehydes; protein oxidation alters folding, catalytic activity, and degradation pathways; and oxidative DNA damage impairs genomic stability. Of particular importance is mitochondrial vulnerability. Oxidative modification of mitochondrial proteins and lipids disrupts electron transport chain function, leading to impaired ATP production and further ROS generation. This establishes a positive feedback loop, in which mitochondrial dysfunction both results from and contributes to oxidative stress, ultimately driving energetic failure and neuronal death [10,17].

Dopaminergic neurons are uniquely susceptible to this process due to their high metabolic demand, autonomous pacemaking activity, and reliance on calcium-dependent signalling, all of which increase mitochondrial workload and ROS production. In addition, their exposure to dopamine-derived redox chemistry places them at a biochemical disadvantage relative to other neuronal populations. This convergence of metabolic and chemical stressors defines the concept of selective neuronal vulnerability in PD [17].

Dopamine-derived oxidation products may also contribute directly to neuroimmune signalling. In experimental systems, aminochrome induces microglial and astrocytic activation, including in primary cultures, organotypic midbrain preparations, and in vivo models, supporting a mechanistic link between dopamine oxidation and neuroinflammatory responses [91,92,93].

Oxidative stress also serves as a potent initiator of neuroinflammatory signalling. Damaged neurons release danger-associated molecular patterns, oxidised lipids, and protein aggregates that activate microglia through pattern-recognition receptors. Activated microglia, in turn, produce pro-inflammatory cytokines (e.g., TNF-α and IL-1β), nitric oxide, and additional ROS, thereby amplifying the oxidative environment. Nitric oxide can react with superoxide to form peroxynitrite, a highly reactive species that can nitrate proteins and further impair mitochondrial function. This interplay establishes a bidirectional coupling between oxidative stress and inflammation, in which each process reinforces the other [16].

Experimental models and human studies indicate that this neuroinflammatory response can become chronic and self-sustaining. Exposure to environmental toxins, such as MPTP or rotenone, induces persistent microglial activation that persists beyond the initial insult [94,95]. Similarly, extracellular release of neuromelanin from degenerating neurons acts as a potent immunogenic signal, perpetuating microglial activation and cytokine production [86]. These observations suggest that once initiated, the oxidative–inflammatory axis may operate independently of the original trigger, driving progressive neurodegeneration through feed-forward mechanisms [16].

At the systems level, oxidative stress and inflammation are not isolated phenomena but components of an integrated pathological network. Dopamine oxidation, mitochondrial dysfunction, α-synuclein aggregation, impaired proteostasis, and immune activation converge to create a toxic microenvironment that selectively affects vulnerable neuronal populations [10,16]. For example, oxidative modification of α-synuclein promotes aggregation, while aggregated α-synuclein further impairs mitochondrial and lysosomal function, thereby enhancing oxidative stress and inflammatory signalling [10,29]. This network perspective reframes PD as a disorder of failed cellular resilience and network instability, rather than a purely degenerative process.

From a translational standpoint, these insights necessitate a shift beyond dopamine-centric therapies. Current treatments largely address neurotransmitter deficiency but do not correct the underlying redox imbalance or immune dysregulation [39]. Future strategies should aim to restore homeostasis across this axis, including enhancing endogenous antioxidant systems, stabilising mitochondrial function, modulating redox-active metal ions, and selectively regulating microglial activation [16]. Importantly, such interventions must preserve physiological signalling while attenuating pathological amplification of oxidative and inflammatory pathways.

Parallel advances in biomarker development are beginning to capture these processes in vivo. Molecular imaging of microglial activation [96], quantification of oxidative metabolites in biofluids, and multi-omics profiling [65] offer the potential to track redox and inflammatory dynamics with increasing precision. These approaches may enable earlier disease detection, improved patient stratification, and monitoring of therapeutic response at the level of underlying biology [75].

In summary, oxidative stress and neuroinflammation represent closely interacting processes that integrate metabolic chemistry, mitochondrial dysfunction, and immune signalling within a common pathological framework in Parkinson’s disease [16,83]. Their strong mechanistic rationale has generated substantial therapeutic interest, and antioxidant, metal-modulating, mitochondrial, and anti-inflammatory approaches have shown neuroprotective effects in numerous experimental models. However, clinical translation has been inconsistent, and no broadly targeted antioxidant or anti-inflammatory strategy has yet demonstrated robust disease-modifying efficacy in PD [16,39]. This translational gap may reflect insufficient CNS target engagement, intervention after substantial neuronal loss has already occurred, heterogeneity in the contribution of redox and inflammatory mechanisms among patients, and the physiological roles of ROS and inflammatory signalling in normal cellular function. Thus, although the oxidative stress–neuroinflammation axis remains an important mechanistic target for investigation, successful therapeutic modulation will likely require pathway-specific interventions, appropriate patient selection, and clear evidence of target engagement rather than nonspecific suppression of oxidative or inflammatory processes.

### 5.4. Reactive Nitrogen Species and Regulation of Dopamine Levels

Reactive nitrogen species (RNS) define a critical biochemical interface between neuroinflammation, redox chemistry, and dopaminergic regulation, linking immune activation directly to enzymatic control of dopamine synthesis. In the inflamed brain, activation of microglia induces expression of inducible nitric oxide synthase (iNOS), driving sustained production of nitric oxide (NO) alongside cytokines and chemokines that reshape the local microenvironment. Although transient NO signalling participates in physiological processes, prolonged iNOS activation establishes a nitrosative milieu that profoundly alters neuronal function and viability [6,16].

At the chemical level, the pathogenic potential of NO emerges from its rapid reaction with superoxide (O_2_•^−^) to form peroxynitrite (ONOO^−^), a highly reactive oxidant with both nitrating and oxidising capacity. This reaction effectively couples mitochondrial ROS production with inflammatory NO signalling, generating a species capable of lipid peroxidation, protein nitration, and mitochondrial impairment. Peroxynitrite can further decompose into secondary radicals, including hydroxyl and nitrogen dioxide species, amplifying oxidative damage and propagating a self-reinforcing redox cascade [17].

Dopaminergic neurons are particularly susceptible to this chemistry due to their high basal oxidative load and reliance on tightly regulated redox balance. Within these cells, peroxynitrite and related RNS directly target key components of dopamine metabolism. A central node of this regulation is TH, whose activity is exquisitely sensitive to post-translational modification. Peroxynitrite induces tyrosine nitration within TH, altering its structural and catalytic properties and reducing enzymatic activity [97]. In parallel, RNS can modify cysteine residues through S-thiolation, which inhibits TH activity, whereas NO-mediated S-nitrosylation has been reported to enhance TH activity, illustrating the context-dependent effects of nitrosative modifications on dopamine synthesis [98,99]. These modifications collectively impair dopamine synthesis, establishing a direct mechanistic link between inflammatory signalling and neurotransmitter deficiency.

Beyond TH, RNS influence multiple layers of dopaminergic regulation. Nitrosative modification of mitochondrial proteins impairs electron transport chain function, increasing ROS production and reinforcing oxidative stress [17]. Concurrently, nitration and oxidation of proteins involved in vesicular transport and dopamine handling—such as VMAT2 and cytoskeletal elements—can disrupt intracellular compartmentalisation, increasing cytosolic dopamine exposure and further promoting oxidative chemistry [100,101]. Thus, RNS act not only on synthesis but across the entire dopaminergic lifecycle, amplifying vulnerability at multiple nodes.

An additional layer of control is provided by DJ-1 (PARK7), a redox-sensitive protein that functions as a molecular sensor and regulator of oxidative stress. DJ-1 modulates TH expression through transcriptional and epigenetic mechanisms, including regulation of chromatin acetylation at the TH promoter [102]. Importantly, DJ-1 activity is itself governed by oxidation of key cysteine residues, enabling it to respond dynamically to changes in intracellular redox state [28]. Under physiological conditions, this system may serve an adaptive role: increased oxidative or nitrosative stress suppresses TH activity, thereby limiting dopamine synthesis and reducing further generation of reactive species. However, in chronic disease states, sustained DJ-1 activation and TH suppression contribute to a progressive decline in dopaminergic function, transforming an initially protective response into a maladaptive driver of neurotransmitter deficiency [10,15].

From a systems perspective, RNS-mediated regulation of dopamine represents a dynamic balance between neuroprotection and metabolic compromise. Transient inhibition of dopamine synthesis may reduce oxidative burden during acute stress, but persistent nitrosative signalling disrupts homeostasis, impairing neuronal function and promoting degeneration [97,100]. This duality reflects a broader principle in redox biology: the same chemical pathways that confer adaptability under physiological conditions can drive pathology when chronically activated.

Importantly, RNS signalling is tightly integrated with neuroinflammatory processes. Activated microglia not only generate NO but also produce cytokines that modulate neuronal signalling pathways and alter gene expression. In turn, damaged neurons release signals that further activate microglia, establishing a bidirectional feedback loop between inflammation and redox imbalance. Within this loop, RNS serve as both mediators and amplifiers, linking immune activation to metabolic dysfunction [16].

These findings have potential translational implications, but their clinical relevance remains uncertain. Current therapeutic approaches in Parkinson’s disease largely focus on restoring dopaminergic function and do not directly target nitrosative stress or its effects on dopamine metabolism [39]. Experimental studies suggest that modulation of iNOS activity, limitation of peroxynitrite formation, preservation of TH structural integrity, and stabilisation of mitochondrial function may reduce nitrosative injury, while DJ-1-dependent pathways represent an additional mechanism through which adaptive redox responses could potentially be supported [16,28,97]. However, these pathways also have important physiological signalling functions, and sufficiently selective modulation within the human brain remains challenging. At present, there is no clinical evidence establishing direct targeting of iNOS, peroxynitrite, or DJ-1 as a disease-modifying strategy in PD. These mechanisms should therefore be considered promising candidates for further experimental and clinical validation rather than established therapeutic targets.

Nitrosative modifications, including protein nitration and S-nitrosylation, may also provide mechanistically informative biomarkers of redox stress. However, their specificity, temporal relationship to neurodegeneration, and ability to predict progression or treatment response in PD remain insufficiently validated.

In summary, reactive nitrogen species represent a central regulatory axis linking inflammation, redox chemistry, and dopamine metabolism [16,97]. Their effects extend from direct chemical modification of enzymes to systems-level disruption of neuronal homeostasis. Understanding this axis at the intersection of structural biology and chemical reactivity provides a framework for investigating how nitrosative stress contributes to dopaminergic dysfunction and for evaluating whether these pathways can be safely and selectively targeted in clinically relevant settings. It underscores the need to integrate redox biology into the core conceptual model of Parkinson’s disease.

## 6. Cellular Models of Dopaminergic Biology

Experimental cell models have been central to dissecting dopamine metabolism, yet they remain constrained by an inherent limitation: dopaminergic function is a context-dependent phenomenon shaped by cellular identity, subcellular organisation, and microenvironmental chemistry. Consequently, widely used in vitro systems often fail to reproduce the integrated metabolic, electrophysiological, and redox properties of human midbrain dopaminergic neurons, particularly in the context of PD [10,31].

Early insights into catecholamine biosynthesis and enzymatic regulation were derived from immortalised cell lines, which offered experimental accessibility but limited physiological fidelity. PC12 cells, originating from rat adrenal pheochromocytoma, have been instrumental in characterising TH kinetics, vesicular storage, and catecholamine release [103]. However, their non-neuronal lineage and tumour-derived metabolic state limit their relevance, particularly for modelling neuronal redox balance and mitochondrial dynamics. Similarly, MN9D cells, generated by fusing embryonic mesencephalic neurons with neuroblastoma cells, exhibit partial dopaminergic features but lack the electrophysiological maturity and calcium-dependent pacemaking activity that define substantia nigra neurons [104]. These deficiencies limit their capacity to model activity-dependent dopamine metabolism and the coupling of oxidative stress.

The widely used SH-SY5Y neuroblastoma line illustrates the broader challenges of cancer-derived models. Although these cells can be chemically differentiated into neuron-like phenotypes, their catecholaminergic identity is heterogeneous and unstable, often skewing toward noradrenergic rather than dopaminergic profiles. Variability in the expression of key enzymes—including TH, AADC, and VMAT2—further undermines reproducibility [105]. At a chemical level, such variability disrupts the stoichiometric balance between synthesis, storage, and degradation, which is essential for maintaining physiological dopamine homeostasis.

More advanced systems, such as LUHMES (Lund human mesencephalic) cells, represent a significant step toward physiological relevance. Derived from human foetal ventral mesencephalon, LUHMES cells can be differentiated into postmitotic neurons that express dopaminergic markers, exhibit neurite architecture, and exhibit electrophysiological activity [106]. Importantly, these cells display a more faithful integration of dopamine synthesis, vesicular compartmentalisation, and mitochondrial metabolism, making them suitable for studying redox-dependent vulnerability. Nevertheless, they remain reductionist monocultures, lacking the multicellular interactions and extracellular constraints that define the in vivo brain environment.

The emergence of iPSC-derived dopaminergic neurons has transformed the field by enabling the generation of patient-specific, genetically defined cellular models. These systems recapitulate key aspects of human dopaminergic biology, including susceptibility to mitochondrial dysfunction, α-synuclein aggregation, and dopamine-mediated oxidative stress [10]. Crucially, iPSC-derived neurons retain disease-associated genetic backgrounds, allowing investigation of how genotype, metabolism, and environmental stressors converge on cellular phenotype. This approach provides a platform for both mechanistic studies and precision medicine applications [31].

In parallel, differentiation of human embryonic stem cells into dopaminergic neurons has demonstrated functional integration in animal models, including restoration of motor behaviour following transplantation [107]. These findings highlight the potential of stem cell-based systems not only for modelling disease but also for cell replacement therapies. However, ethical considerations, scalability, and variability in differentiation efficiency remain challenges that limit widespread application.

Despite these advances, a central limitation persists: most in vitro systems lack the multicellular and spatial complexity required to reproduce dopaminergic physiology. In vivo, dopamine metabolism is distributed across neurons, astrocytes, microglia, and vascular elements, with each component contributing to substrate flux, redox buffering, and inflammatory signalling. Two-dimensional monocultures fail to capture these interactions, as well as the three-dimensional architecture, synaptic connectivity, and dynamic neuromodulatory inputs that shape neuronal behaviour [108].

Emerging technologies are beginning to address these limitations. Three-dimensional brain organoids, co-culture systems incorporating neurons and glia, and microfluidic platforms (“organ-on-chip”) enable reconstruction of more physiologically relevant microenvironments [108,109]. These systems allow spatial segregation of cell types, controlled gradients of metabolites, and real-time analysis of intercellular interactions. From a chemical perspective, they provide an opportunity to study dopamine metabolism within defined microdomains, capturing the interplay between synthesis, oxidation, and detoxification under conditions that more closely approximate in vivo physiology.

Integration of these advanced models with multi-omics approaches, high-resolution imaging, and computational modelling further enhances their utility. Single-cell transcriptomics can resolve heterogeneity in enzyme expression and redox pathways [74], while metabolomics enables quantitative mapping of dopamine-derived intermediates and oxidative products [65]. Together, these approaches transform experimental systems from static models into dynamic, systems-level platforms capable of capturing the complexity of dopaminergic networks.

From a translational perspective, these considerations underscore the need for a paradigm shift. Rather than relying on simplified models that isolate individual components, future research must prioritise human-relevant, multicellular, and context-aware systems that reflect the integrated nature of dopamine metabolism [31,109]. Such models will be essential for identifying mechanism-based therapeutic targets, evaluating drug responses, and bridging the gap between experimental findings and clinical application.

In summary, cellular models of dopaminergic biology have evolved from reductionist systems toward increasingly sophisticated platforms that capture structural, metabolic, and redox complexity. However, no single model fully recapitulates the in vivo environment. Advancing this field will require integration of complementary systems, combining the experimental tractability of in vitro models with the physiological relevance of human-derived and multicellular platforms. Only through such an approach can the full complexity of dopamine biology be understood and effectively translated into disease-modifying therapies.

## 7. From the Laboratory to Clinical Application

Despite substantial advances in molecular neuroscience, Parkinson’s disease remains a disorder in which mechanistic understanding has outpaced therapeutic translation. Current diagnostic frameworks rely predominantly on clinical phenotyping supported by neuroimaging modalities—such as DAT imaging and advanced MRI techniques—which enable classification of established disease but lack the sensitivity to detect preclinical metabolic dysfunction. This limitation reflects a deeper challenge: the absence of biomarkers that capture the earliest perturbations in dopamine synthesis, compartmentalisation, and redox homeostasis, which precede irreversible neuronal loss [39,75,110].

Therapeutically, the field remains centred on dopamine replacement, with levodopa combined with a peripheral AADC inhibitor, such as carbidopa, remaining the most effective symptomatic treatment for PD. By providing exogenous L-DOPA downstream of TH, this approach increases dopamine synthesis in surviving dopaminergic neurons and, as nigrostriatal denervation progresses, in other cellular populations capable of AADC-mediated decarboxylation. From a biochemical perspective, increased cytosolic catechol availability can provide additional substrate for spontaneous oxidation and MAO-mediated metabolism, potentially generating quinones, DOPAL, and reactive oxygen species under experimental conditions [6,45]. However, these biochemical and preclinical observations do not demonstrate that therapeutic levodopa produces clinically relevant oxidative neurotoxicity or accelerates nigrostriatal degeneration in patients.

The potential neurotoxicity of levodopa has been debated for decades, in part because L-DOPA and dopamine can generate reactive species in biochemical and experimental systems. Clinical evidence, however, has not demonstrated a detrimental effect of levodopa on PD progression. In the ELLDOPA trial, clinical outcomes did not support accelerated disease progression with levodopa, although the accompanying dopamine-transporter imaging findings were difficult to reconcile with the clinical results [111]. The delayed-start LEAP trial subsequently found no evidence of either a beneficial or detrimental disease-modifying effect of early levodopa treatment over 80 weeks [112], and its five-year follow-up likewise found no significant difference in disease progression between early- and delayed-start groups [113]. Thus, levodopa-associated oxidative chemistry remains mechanistically relevant to dopamine biology, but its contribution to progressive neurodegeneration in treated patients is not supported by current clinical evidence.

Dopamine metabolism under pharmacological conditions can nevertheless have measurable consequences. COMT-dependent metabolism of L-DOPA and dopamine consumes S-adenosyl-L-methionine (SAM) and can increase homocysteine formation [114]. Motor fluctuations and dyskinesias are also well-established complications of chronic levodopa therapy, particularly as disease duration, nigrostriatal denervation, and pulsatile dopaminergic stimulation increase [39]. These complications are multifactorial and should not be interpreted as evidence of levodopa-induced neuronal toxicity.

Adjunctive pharmacological strategies primarily aim to optimise levodopa pharmacokinetics and dopaminergic stimulation. MAO-B inhibitors reduce dopamine degradation, whereas COMT inhibitors prolong levodopa availability by limiting its peripheral metabolism [39]. Folate and vitamin B12 supplementation may help mitigate treatment-associated elevations in homocysteine in selected patients [114], while continuous levodopa infusion and other continuous dopaminergic delivery approaches aim to reduce fluctuations in plasma and brain dopamine availability. These interventions improve symptomatic control but have not established disease-modifying effects, underscoring the distinction between optimisation of dopaminergic replacement and modification of the underlying neurodegenerative process.

Beyond modulation of dopamine synthesis and degradation, preservation of detoxification capacity represents another mechanistically relevant therapeutic concept. The ALDH–DOPAL axis is of particular interest because impaired clearance of the reactive aldehyde DOPAL could increase electrophilic and oxidative stress in vulnerable dopaminergic neurons. Experimental enhancement of ALDH activity or limitation of DOPAL accumulation has therefore been proposed as a potential protective strategy, although selective and clinically viable approaches remain undeveloped and the causal contribution of this pathway to human PD has not been established. Similarly, dopamine-derived metabolite ratios and aldehyde-related signatures may provide mechanistically informative biomarkers, but their clinical utility is limited by analytical challenges, compartment specificity, and the difficulty of measuring reactive intermediates such as DOPAL in vivo.

Increasing attention has therefore shifted toward non-dopaminergic and systems-level interventions. Modulation of glutamatergic, serotonergic, and adrenergic pathways offers avenues to address both motor and non-motor symptoms, while deep brain stimulation provides substantial clinical benefit through circuit-level modulation [39]. However, these strategies do not alter the progressive metabolic and cellular dysfunction that underlies neuronal degeneration. Similarly, anti-inflammatory and neuroprotective approaches—including modulation of microglial activation, oxidative stress, quinone-detoxifying systems such as NQO1 and GSTM2, or nicotinic receptor signalling—have demonstrated promise in experimental models but have yet to yield consistent clinical efficacy [16,67,68].

Regenerative and gene-based therapies represent a more ambitious attempt to reconstruct dopaminergic function at its source. Transplantation of iPSC-derived dopaminergic neurons offers the possibility of patient-specific cell replacement, with early studies demonstrating functional integration and symptomatic improvement [115,116]. However, challenges related to cell survival, synaptic integration, tumourigenicity, and long-term stability remain significant [117].

In parallel, viral vector-mediated gene therapies targeting enzymes of dopamine biosynthesis—such as AADC, TH, and GTPCH—have entered clinical evaluation [47,118]. AADC gene delivery enhances local conversion of levodopa to dopamine, effectively increasing enzymatic capacity within the striatum [47]. However, such approaches often address single nodes within a complex metabolic network and may therefore not fully restore the spatiotemporal regulation of dopamine synthesis, cofactor availability, and redox balance. More comprehensive strategies that reconstitute coordinated biosynthetic function or preserve enzyme regulation, cofactor availability, and vesicular coupling may offer greater physiological fidelity, but they introduce additional technical and ethical complexity [118].

These developments collectively point toward a necessary conceptual transition. Although dopamine deficiency remains central to the motor manifestations and symptomatic treatment of PD, the underlying neurodegenerative process involves broader disturbances in dopamine synthesis, compartmentalisation, degradation, redox chemistry, mitochondrial function, and neuroimmune signalling [6,16]. Within this framework, effective therapies must move beyond replacement toward restoration of homeostasis across interconnected biochemical and cellular domains.

Accordingly, restoration of dopaminergic signalling and preservation of metabolic homeostasis should be viewed as complementary therapeutic objectives. In advanced disease, where substantial nigrostriatal denervation has already occurred, optimisation of the remaining metabolic network alone is unlikely to restore normal dopaminergic function, and dopamine replacement, circuit-based therapies, or regenerative approaches remain necessary. By contrast, strategies aimed at preserving compartmentalisation, detoxification capacity, mitochondrial integrity, and redox balance may be most relevant when applied before extensive neuronal loss.

From a translational perspective, this shift requires integration across multiple scales. Molecular biomarkers reflecting dopamine metabolism, oxidative stress, and inflammation must be developed to enable early detection and patient stratification [75]. Advanced imaging modalities should be leveraged to map regional and temporal changes in dopaminergic function [73,110]. At the same time, precision medicine approaches that incorporate genetic, metabolic, and environmental data will be essential for tailoring interventions to individual disease trajectories [4].

From a scientific standpoint, bridging the gap between laboratory insights and clinical applications requires a deeper understanding of dopamine biology as an integrated network rather than a linear pathway [6]. This includes elucidating the structural and chemical determinants of enzyme function, the dynamics of intracellular compartmentalisation, and the interplay between metabolism and immune signalling.

In summary, translating dopaminergic biology into clinical practice remains incomplete because current therapeutic strategies largely compensate for dopamine deficiency without addressing the broader biochemical and cellular processes that govern dopaminergic homeostasis. Figure 4 integrates these concepts into a systems-level model, contrasting the coordinated regulation of dopamine synthesis, vesicular storage, degradation, detoxification, redox buffering, mitochondrial function, and neuroimmune signalling in the healthy neuron with their progressive disruption during neurometabolic failure in Parkinson’s disease. This framework highlights dopamine deficiency as part of a wider collapse of metabolic resilience and identifies redox balance, intracellular compartmentalisation, detoxification capacity, mitochondrial integrity, and neuroimmune homeostasis as mechanistically relevant intervention points whose disease-modifying potential requires further clinical validation. A future therapeutic paradigm centred on restoring this integrated metabolic balance, rather than solely replacing dopamine, may therefore provide a more rational path toward preserving neuronal resilience and altering disease progression.

## 8. Discussion

The emerging view of dopamine metabolism as a chemically constrained and highly regulated network exposes a fundamental tension within the field. Although structural and mechanistic information on individual enzymes, transporters, and regulatory interactions has expanded substantially, integration of these findings into a coherent cellular and translational framework remains incomplete. This gap is not merely technical but conceptual, reflecting the difficulty of connecting molecular mechanisms with the spatial, metabolic, and redox complexity of dopaminergic systems [6].

A central challenge lies in the fragmentation of dopamine biology across disciplinary boundaries. Enzymology, structural biology, neurochemistry, and clinical neuroscience have often addressed individual components of the pathway separately. Consequently, the extent to which enzymatic reactions, cofactor availability, vesicular transport, and degradation are spatially coordinated in intact dopaminergic neurons remains incompletely understood [6,14]. In particular, proposed mechanisms such as substrate channelling or local metabolic coupling should be distinguished from the established sequential dependence of these pathways. Quantitative models integrating enzyme kinetics, intracellular compartmentalisation, redox dynamics, and cellular heterogeneity will be required to determine whether higher-order spatial organisation materially influences dopamine metabolism under physiological or pathological conditions.

A second major limitation concerns the structural resolution of dopamine metabolic machinery in its native cellular context. High-resolution structures have defined the conformational and catalytic properties of individual components, including TH, MAO, COMT, AADC, and VMAT2 [8,12,41,49,56], while specific protein–protein interactions involving selected components have also been demonstrated experimentally. These findings establish structural regulation at the level of individual proteins and particular molecular interactions. By contrast, direct evidence that dopamine-metabolising enzymes and transporters assemble into stable higher-order multi-enzyme complexes or discrete functional microdomains in dopaminergic neurons remains limited. Likewise, direct substrate channelling between these components has not been demonstrated in native mammalian or human dopaminergic neurons [14]. Spatial coupling and higher-order organisation should therefore be regarded as mechanistically plausible models rather than established features of dopamine metabolism. Emerging approaches such as cryo-electron tomography and other in situ structural methods may provide the spatial resolution required to test these hypotheses directly [119].

Equally important is the limited understanding of cofactor dynamics as regulatory determinants. Molecules such as BH_4_, FAD, and SAM are often treated as static participants in enzymatic reactions, yet their intracellular concentrations, redox states, and spatial distribution fluctuate dynamically. These fluctuations are likely to exert first-order control over metabolic flux, particularly under conditions of stress or disease [6,19]. However, real-time measurement of cofactor availability and turnover within specific neuronal compartments remains technically challenging, representing a critical barrier to understanding how metabolic regulation is achieved in vivo.

Another unresolved issue is the chemical fate of reactive intermediates, particularly quinones and aldehydes such as DOPAL. While their formation and toxicity are well established, their spatiotemporal dynamics, interaction networks, and threshold levels for cellular damage remain poorly quantified [18,20]. Current models often assume linear accumulation of toxic species, yet it is increasingly evident that non-linear, threshold-dependent behaviours—driven by feedback loops between oxidation, mitochondrial dysfunction, and proteostasis—govern the transition from physiological to pathological states [10]. Defining these thresholds will be essential for identifying early intervention points and for distinguishing adaptive from maladaptive responses.

The role of cellular heterogeneity and intercellular coupling represents an additional layer of complexity that remains insufficiently resolved. Dopamine metabolism is distributed across neurons, astrocytes, microglia, and vascular elements, each contributing distinct enzymatic activities and redox buffering capacities [6,74]. However, most experimental systems continue to rely on monocultures or simplified models that fail to capture this multicellular integration. As a result, critical processes—such as glial regulation of extracellular dopamine, immune-mediated modulation of redox balance, and metabolic exchange between cell types—are incompletely understood. Bridging this gap will require multicellular and spatially resolved models capable of reproducing the physiological architecture of dopaminergic circuits [108].

A related limitation concerns the uneven strength of evidence across the mechanisms discussed in this Review. Alterations in dopamine metabolism in human PD are supported by post-mortem neurochemical and expression studies, imaging, genetic associations, and selected biofluid measurements, but these approaches provide limited information on dynamic intracellular processes and often cannot establish causality. By contrast, much of the mechanistic evidence concerning DOPAL toxicity, aminochrome-mediated injury, redox cycling, altered cofactor dynamics, and proposed spatial coupling of metabolic enzymes derives from biochemical, cellular, and animal models. These experimental systems are essential for defining plausible mechanisms but should not be interpreted as direct demonstrations of their quantitative contribution to human PD.

Ageing provides an important biological context for these mechanisms. Several processes implicated in PD—including mitochondrial dysfunction, reduced proteostatic and autophagy–lysosomal capacity, declining redox resilience, altered iron homeostasis, and chronic low-grade neuroinflammatory signalling—also emerge progressively during normal ageing [120,121,122,123]. Ageing is likewise accompanied by measurable changes in dopaminergic function, including reductions in dopamine transporter and receptor availability and evidence of declining dopamine storage capacity, although dopamine synthesis capacity may be relatively preserved [124,125]. These parallels should not be interpreted as indicating that PD represents simply an accelerated form of ageing. Rather, age-related reductions in metabolic and cellular resilience may lower the threshold at which additional genetic, environmental, or biochemical disturbances become sufficient to produce sustained dopaminergic dysfunction and neurodegeneration [120,121]. Within this framework, ageing can be viewed as a background of progressively diminished homeostatic reserve that increases the susceptibility of nigrostriatal neurons to PD-associated stressors.

Translationally, one of the most pressing challenges is the lack of sensitive, mechanism-based biomarkers. Current diagnostic tools detect relatively late-stage changes, such as neuronal loss or altered dopamine transporter availability, but do not capture early perturbations in dopamine synthesis, compartmentalisation, or redox homeostasis [75,110]. Biomarkers reflecting dynamic processes—such as cofactor depletion, protein nitration, aldehyde accumulation, or altered vesicular storage—remain largely unexplored in clinical settings. The development of such markers will be essential for enabling preclinical diagnosis, patient stratification, and monitoring of therapeutic response [75].

A further limitation lies in the disconnect between therapeutic strategies and underlying biochemical mechanisms. Existing treatments primarily target dopamine levels rather than the processes governing its safe handling [39]. This approach overlooks the central role of oxidative and nitrosative stress, mitochondrial dysfunction, and enzyme dysregulation in driving disease progression [10,16]. Emerging therapies—including gene delivery, stem cell transplantation, and enzyme modulation—represent important advances [47,116,118], yet they often address isolated components of the system rather than restoring network-level homeostasis. Future interventions must therefore be designed with an explicit understanding of dopamine metabolism as an interconnected system, in which modulation of one node inevitably affects others.

Finally, there is a broader conceptual gap in how Parkinson’s disease itself is defined. The traditional view of PD as a disorder of dopamine deficiency is increasingly insufficient to account for the multifactorial and dynamic nature of dopaminergic dysfunction. A complementary framework views PD as involving a progressive failure of neurometabolic resilience, in which disturbances in redox balance, protein homeostasis, mitochondrial function, and immune signalling interact with the loss of dopaminergic neurotransmission and contribute to the vulnerability of nigrostriatal neurons [16,17]. This perspective shifts the focus from replacement to restoration, emphasising the need to stabilise the underlying biochemical network rather than compensating for its failure.

To provide a more explicit framework for future research, Table 4 summarises the principal knowledge gaps identified throughout this Review and the key questions that should guide subsequent studies in the field.

Looking forward, addressing these challenges will require convergence across disciplines and scales. Structural biology must be integrated with live-cell imaging and metabolomics to determine whether, and to what extent, higher-order spatial organisation contributes to dopamine metabolism in intact cells. Systems biology approaches should be employed to construct predictive models that incorporate enzyme kinetics, compartmental dynamics, and feedback regulation. Human-relevant experimental systems, including iPSC-derived neurons and organoid models, must be further developed to capture genetic and environmental variability [31,109]. Progress at the experimental level will depend on technologies capable of directly resolving dopamine metabolic flux, redox chemistry, intracellular compartmentalisation, and cellular interactions in human-relevant systems.

In parallel, clinical research will need to integrate molecular biomarkers, advanced imaging, and computational analysis to improve patient stratification and guide personalised therapeutic strategies [75,110]. Such approaches will also be necessary to establish whether mechanistically informed interventions can influence disease trajectory in addition to improving symptomatic control.

In summary, the study of dopamine metabolism stands at a critical juncture. The field has moved beyond linear pathways to recognise a complex, adaptive, and chemically constrained network [6], yet key gaps remain in structural resolution, dynamic measurement, and translational integration. Addressing these challenges will be essential to determine how system-level biochemical dysfunction contributes to dopaminergic degeneration and to complement established dopamine-centred approaches with mechanism-based strategies aimed at preserving neuronal function and resilience.

## Figures and Tables

**Figure 1:**
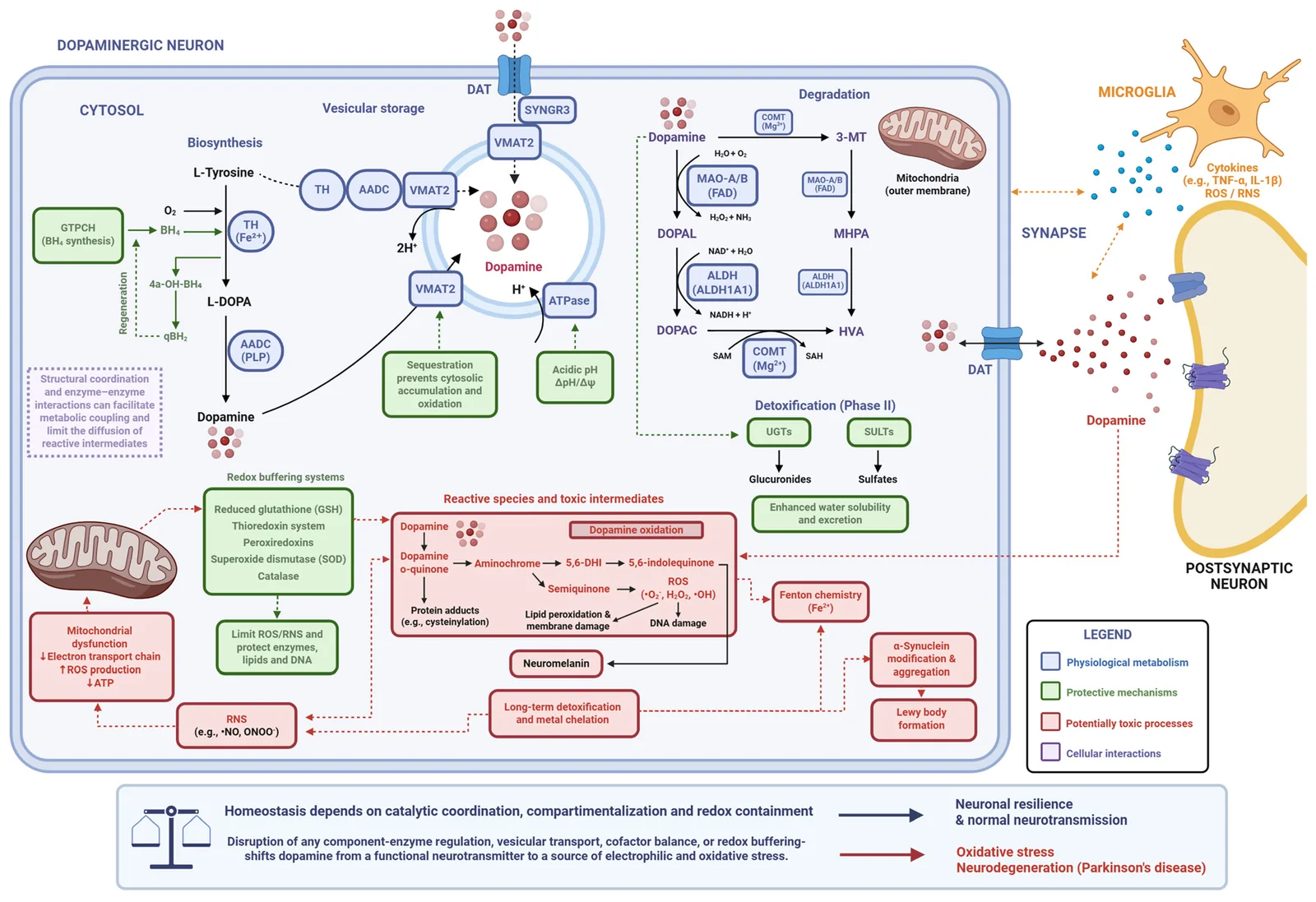
Dopamine metabolism as a chemically constrained and functionally integrated network in dopaminergic neurons. Dopamine metabolism is represented as an interconnected system comprising biosynthesis, vesicular sequestration, synaptic transmission and reuptake, enzymatic degradation, conjugation, redox buffering, and neuron–glia interactions. De novo tetrahydrobiopterin (BH_4_) synthesis is initiated by GTP cyclohydrolase I (GTPCH), while BH_4_ consumed during tyrosine hydroxylase (TH) catalysis is regenerated through the pterin-recycling pathway. TH converts L-tyrosine to L-3,4-dihydroxyphenylalanine (L-DOPA) in an O_2_-, Fe^2+^-, and BH_4_-dependent reaction, followed by aromatic L-amino acid decarboxylase (AADC)-mediated formation of dopamine. Newly synthesised cytosolic dopamine is transported into synaptic vesicles by vesicular monoamine transporter 2 (VMAT2), an H^+^/monoamine antiporter driven by the proton electrochemical gradient generated by vesicular V-type H^+^-ATPase (V-ATPase). In addition to this classical cytosolic route, experimentally supported associations among TH-, AADC-, and VMAT2-containing vesicles may facilitate local coupling between dopamine synthesis and vesicular sequestration. Following release, extracellular dopamine is recaptured by the dopamine transporter (DAT); interactions involving DAT, synaptogyrin-3 (SYNGR3), and VMAT2 may similarly facilitate coupling between reuptake and vesicular refilling. These local arrangements are represented as functional coupling models and do not imply obligatory substrate channelling. Cytosolic dopamine can be degraded through two principal enzymatic sequences. In the MAO-first route, monoamine oxidase A/B (MAO-A/B), located at the outer mitochondrial membrane, converts dopamine to 3,4-dihydroxyphenylacetaldehyde (DOPAL), with formation of H_2_O_2_ and ammonia; DOPAL is subsequently oxidised by aldehyde dehydrogenases, including ALDH1A1, to 3,4-dihydroxyphenylacetic acid (DOPAC), which is methylated by catechol-O-methyltransferase (COMT) to homovanillic acid (HVA). In the COMT-first route, dopamine is converted to 3-methoxytyramine (3-MT), followed by MAO-mediated formation of 3-methoxy-4-hydroxyphenylacetaldehyde (MHPA) and ALDH-dependent conversion to HVA. Sulphation and glucuronidation provide additional Phase II conjugation pathways contributing to catechol clearance. Alternatively, dopamine can undergo spontaneous or metal-facilitated oxidation to dopamine o-quinone, followed by aminochrome, 5,6-dihydroxyindole (5,6-DHI), and 5,6-indolequinone formation. Quinone/semiquinone redox cycling contributes to reactive oxygen species (ROS) formation, whereas electrophilic quinones can generate protein thiol adducts. ROS promote lipid peroxidation, membrane injury, DNA damage, and mitochondrial dysfunction. Antioxidant systems, vesicular sequestration, metabolic detoxification, and neuromelanin formation contribute to containment of dopamine-derived reactive species, while inflammatory and nitrosative signals from neighbouring microglia can further modify neuronal redox homeostasis. Created in BioRender. Guerra-Crespo, M. (2026) https://BioRender.com/p9wisub.

**Figure 2:**
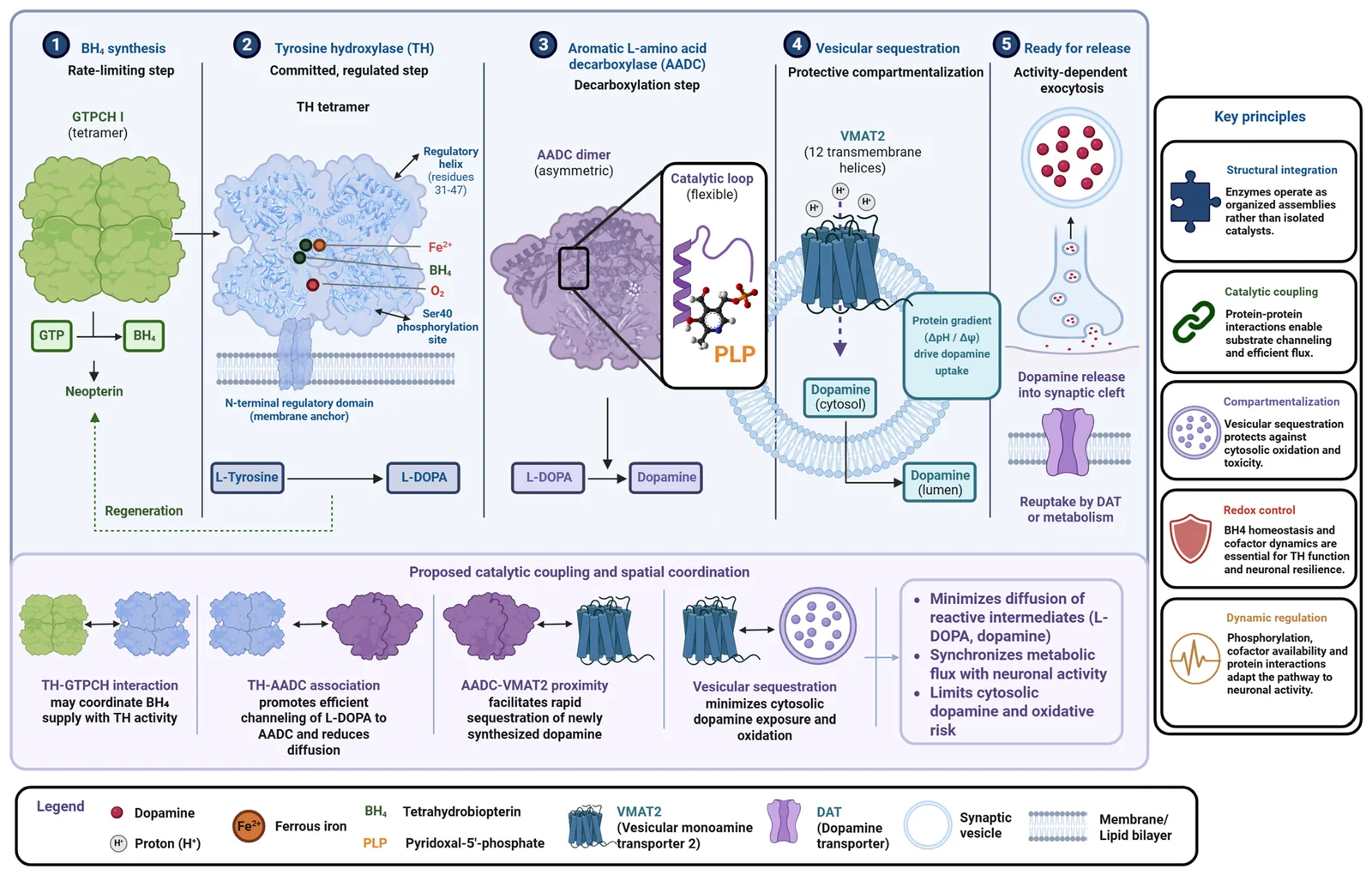
Molecular architecture and spatial organization of dopamine biosynthesis. Dopamine biosynthesis is presented as a structurally coordinated process in which sequential enzymatic reactions are integrated through catalytic coupling, cofactor availability, and intracellular compartmentalization. (1) BH_4_ synthesis: GTP cyclohydrolase I (GTPCH) catalyses the rate-limiting step of tetrahydrobiopterin (BH_4_) biosynthesis, while enzymatic recycling contributes to maintaining cofactor availability during catecholamine synthesis. (2) Tyrosine hydroxylase (TH): TH, a tetrameric non-heme iron enzyme, catalyses the rate-limiting hydroxylation of L-tyrosine to L-3,4-dihydroxyphenylalanine (L-DOPA). Enzymatic activity is regulated by iron and BH_4_ availability, dopamine-mediated feedback inhibition, conformational changes within the N-terminal regulatory domain, and phosphorylation-dependent regulation. (3) Aromatic L-amino acid decarboxylase (AADC): AADC, a pyridoxal 5′-phosphate (PLP)-dependent homodimer, converts L-DOPA into dopamine through decarboxylation, with catalytic-loop dynamics and cofactor-dependent catalysis governing enzymatic efficiency. (4) Vesicular sequestration: Newly synthesized dopamine is rapidly transported into synaptic vesicles by vesicular monoamine transporter 2 (VMAT2) through proton-coupled transport driven by the vesicular electrochemical gradient (ΔpH/Δψ), thereby limiting cytosolic dopamine accumulation and spontaneous oxidation. (5) Ready for release: Vesicularly stored dopamine undergoes activity-dependent exocytosis into the synaptic cleft, after which it can be recaptured by the dopamine transporter (DAT) and returned to intracellular metabolic or vesicular pathways. The lower panel presents a conceptual model in which GTPCH, TH, AADC, VMAT2 and associated regulatory proteins could exhibit functional or spatial coupling. Specific interactions between some of these components have been demonstrated experimentally; however, their organisation into a common biosynthetic microdomain and direct substrate channelling have not been established in dopaminergic neurons. Such organisation could, in principle, coordinate dopamine synthesis with vesicular sequestration and limit exposure to reactive intermediates. Created in BioRender. Guerra-Crespo, M. (2026) https://BioRender.com/m2gwsp4.

**Figure 3:**
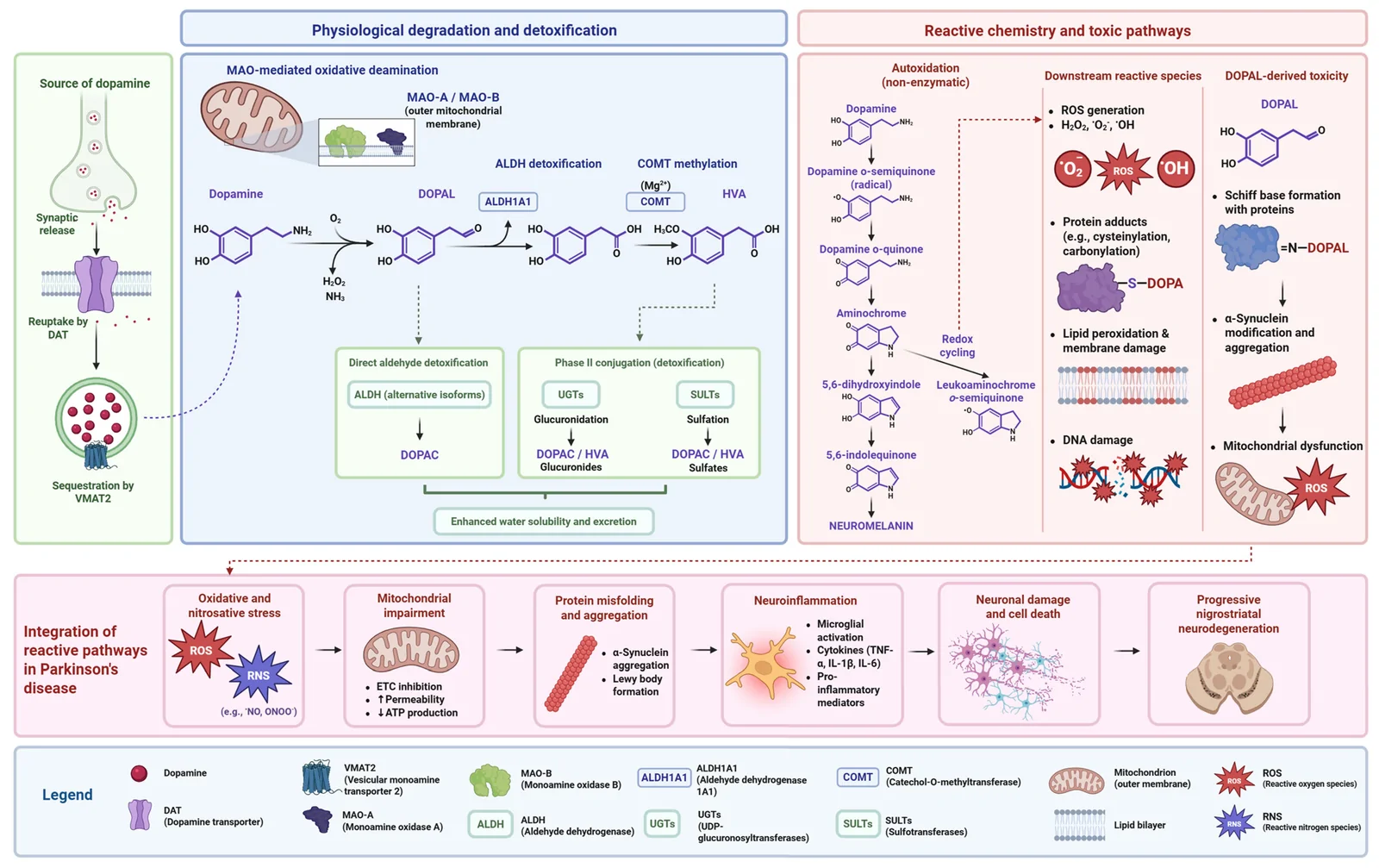
Dopamine degradation, detoxification pathways, and integration of reactive chemistry in Parkinson’s disease. Dopamine metabolism is illustrated as a balance between physiological degradation/detoxification pathways and competing reactions that generate chemically reactive intermediates. Following dopamine transporter (DAT)-mediated reuptake, cytosolic dopamine can be resequestered into synaptic vesicles by vesicular monoamine transporter 2 (VMAT2) or undergo oxidative deamination by monoamine oxidase A and B (MAO-A/MAO-B), located at the outer mitochondrial membrane, generating 3,4-dihydroxyphenylacetaldehyde (DOPAL), hydrogen peroxide (H_2_O_2_), and ammonia. DOPAL is detoxified predominantly by aldehyde dehydrogenase pathways, including ALDH1A1-mediated oxidation to 3,4-dihydroxyphenylacetic acid (DOPAC), which can subsequently be O-methylated by catechol-O-methyltransferase (COMT) to form homovanillic acid (HVA). An alternative degradative route involves prior COMT-mediated methylation of dopamine-derived catechols, whereas additional detoxification occurs through phase II conjugation by UDP-glucuronosyltransferases (UGTs) and sulfotransferases (SULTs), increasing water solubility and facilitating elimination. In parallel, non-enzymatic dopamine autoxidation proceeds through dopamine o-semiquinone radical and dopamine o-quinone to form aminochrome, followed by 5,6-dihydroxyindole and 5,6-indolequinone, which contribute to neuromelanin formation. Aminochrome can also undergo one-electron reduction to leukoaminochrome o-semiquinone, promoting redox cycling and the generation of reactive oxygen species (ROS), including superoxide, hydrogen peroxide, and hydroxyl radical. These oxidation pathways contribute to protein adduct formation (e.g., cysteinylation and carbonylation), lipid peroxidation and membrane damage, and DNA damage. In parallel, DOPAL can form covalent protein adducts, including Schiff-base modifications, promote α-synuclein modification and aggregation, and contribute to mitochondrial dysfunction. The lower panel integrates these pathways into a systems-level framework in which oxidative and nitrosative stress, mitochondrial impairment, protein misfolding and aggregation, neuroinflammation, neuronal damage and cell death, and progressive nigrostriatal neurodegeneration emerge as interconnected consequences of disrupted dopamine homeostasis in Parkinson’s disease. Created in BioRender. Guerra-Crespo, M. (2026) https://BioRender.com/unckpl3.

**Figure 4:**
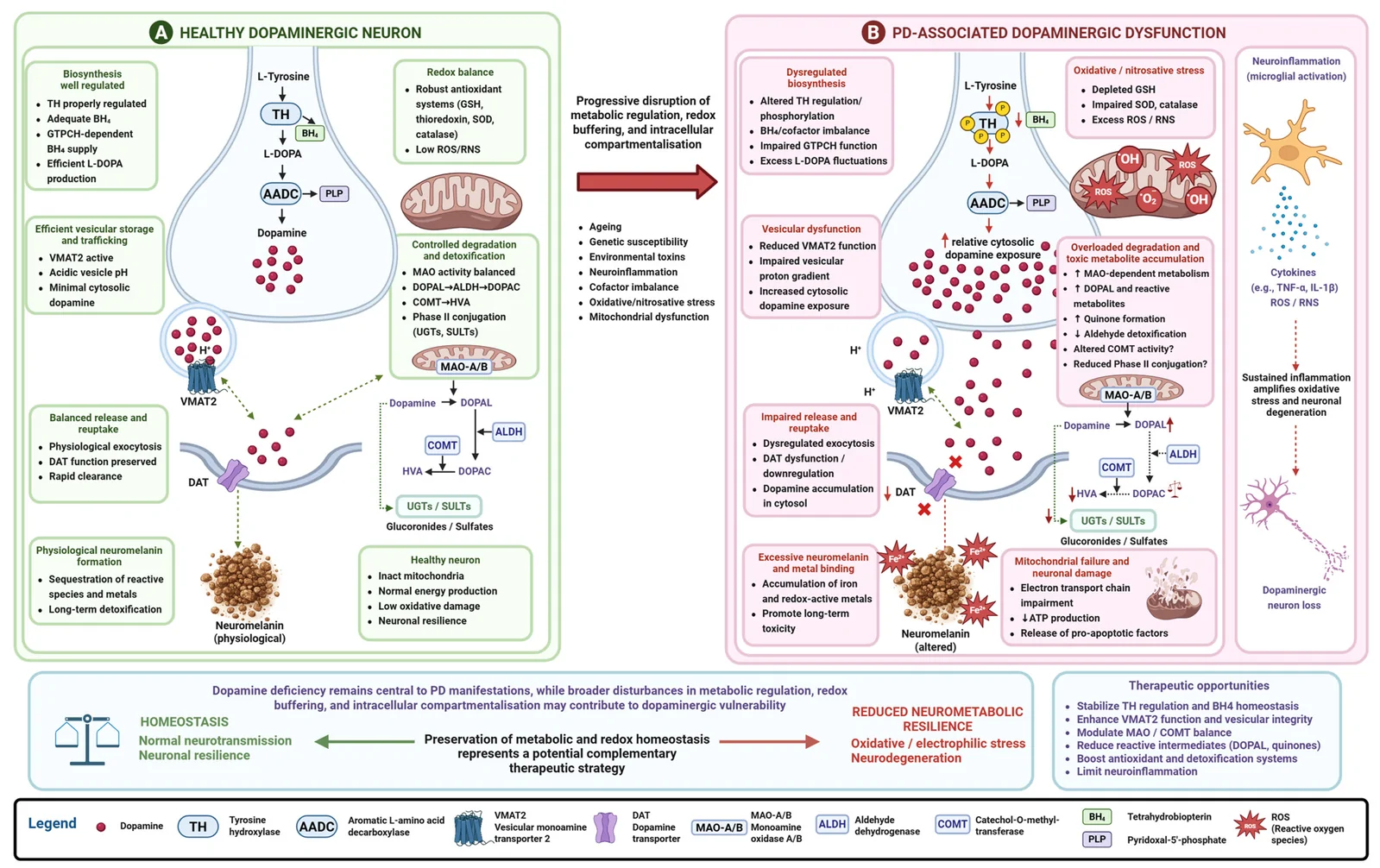
From dopaminergic homeostasis to metabolic and redox dysfunction in Parkinson’s disease. The figure presents an integrative framework contrasting physiological dopamine homeostasis (**A**) with mechanisms that may contribute to dopaminergic dysfunction and vulnerability in Parkinson’s disease (**B**). Under physiological conditions, dopamine synthesis, vesicular sequestration, synaptic release and reuptake, enzymatic degradation, detoxification, redox buffering, mitochondrial function, and neuromelanin-associated sequestration operate together to maintain dopamine availability while limiting cytosolic exposure to reactive intermediates. These processes depend on regulation of tyrosine hydroxylase (TH), tetrahydrobiopterin (BH_4_) availability, aromatic L-amino acid decarboxylase (AADC), vesicular monoamine transporter 2 (VMAT2), dopamine transporter (DAT), monoamine oxidases A and B (MAO-A/MAO-B), aldehyde dehydrogenases (ALDHs), catechol-O-methyltransferase (COMT), and complementary detoxification pathways. In PD, ageing, genetic susceptibility, environmental exposures, mitochondrial dysfunction, cofactor imbalance, oxidative and nitrosative stress, and neuroinflammatory signalling may progressively reduce the capacity of this system to maintain metabolic and redox homeostasis. Impaired vesicular sequestration can increase relative cytosolic dopamine exposure, favouring MAO-dependent metabolism and non-enzymatic oxidation, with formation of reactive aldehydes and quinones. Reduced detoxification capacity, mitochondrial impairment, altered metal and neuromelanin handling, and insufficient antioxidant buffering may further amplify electrophilic and oxidative stress. Neuroinflammatory signalling can interact bidirectionally with these processes and contribute to progressive dopaminergic neuronal dysfunction and loss. Panel (**B**) represents a conceptual integration of mechanisms supported to different extents by biochemical studies, cellular and animal models, human post-mortem tissue, genetic evidence, imaging, and clinical observations; therefore, the depicted alterations should not be interpreted as a single experimentally established sequence occurring uniformly in human PD. Dopamine deficiency remains central to the motor manifestations of PD, while the broader metabolic and redox disturbances illustrated here may contribute to neuronal vulnerability and disease progression. Accordingly, preservation of vesicular function, cofactor and enzyme regulation, aldehyde and quinone detoxification, mitochondrial integrity, redox buffering, and neuroimmune homeostasis represent mechanistically motivated intervention points whose disease-modifying potential requires clinical validation. These approaches should be regarded as complementary to, rather than replacements for, established dopaminergic therapies. Created in BioRender. Guerra-Crespo, M. (2026) https://BioRender.com/pzaif2h.

**Table 1:** Structural and functional overview of the enzymes governing dopamine metabolism.

Enzyme	Cellular Location	Catalytic Reactivation	Substrates/Cofactors	Structural Organization	Main Regulatory Mechanisms	Functional/Pathological Relevance in PD
GTPCH [19]	Cytosol	BH_4_ synthesis	GTP	Decamer	GFRP, phosphorylation	↓ BH_4_
TH [7]	Cytosol	Tyrosine → L-DOPA	Fe^2+^, BH_4_	Tetramer	Dopamine feedback, Ser40 phosphorylation	↓ synthesis
AADC [8]	Cytosol	L-DOPA → Dopamine	PLP	Homodimer	PLP availability	↓ conversion
VMAT2 [6]	Vesicle membrane	Dopamine transport	H^+^ gradient	12 TM helices	ΔpH	Cytosolic dopamine
MAO-A/B [6,20]	Outer mitochondrial membrane	Dopamine oxidation	FAD	Membrane enzyme	Expression/activity	DOPAL formation
ALDH1A1 [20,21]	Cytosol	DOPAL → DOPAC	NAD^+^	Tetramer	Oxidative inhibition	DOPAL accumulation
COMT [22]	Cytosol/glia	O-methylation	Mg^2+^ + SAM	Monomer	Tissue expression	HVA production
UGT/SULT [6]	ER (UGT)/cytosol (SULT)	Conjugation	UDPGA/PAPS	Multiple isoforms	Isoform-specific	Detoxification

AADC, aromatic L-amino acid decarboxylase; ALDH1A1, aldehyde dehydrogenase 1 family member A1; BH_4_, tetrahydrobiopterin; COMT, catechol-O-methyltransferase; DOPAC, 3,4-dihydroxyphenylacetic acid; DOPAL, 3,4-dihydroxyphenylacetaldehyde; ER, endoplasmic reticulum; FAD, flavin adenine dinucleotide; Fe^2+^, ferrous iron; GFRP, GTP cyclohydrolase I feedback regulatory protein; GTP, guanosine triphosphate; GTPCH, GTP cyclohydrolase I; H^+^, proton; HVA, homovanillic acid; L-DOPA, L-3,4-dihydroxyphenylalanine; MAO-A, monoamine oxidase A; MAO-B, monoamine oxidase B; Mg^2+^, magnesium ion; NAD^+^, nicotinamide adenine dinucleotide; PAPS, 3′-phosphoadenosine-5′-phosphosulfate; PD, Parkinson’s disease; PLP, pyridoxal 5′-phosphate; SAM, S-adenosyl-L-methionine; SULT, sulfotransferase; TH, tyrosine hydroxylase; TM, transmembrane; UDPGA, uridine diphosphate glucuronic acid; UGT, UDP-glucuronosyltransferase; VMAT2, vesicular monoamine transporter 2; ΔpH, transmembrane proton gradient; ↓, decreased; →, conversion to.

**Table 2:** Protective versus pathogenic mechanisms controlling dopamine homeostasis.

Protective Mechanism	Physiological Function	Failure Leads to
BH_4_ homeostasis [19]	TH Catalysis	Reduced synthesis
TH regulation [6]	Controlled dopamine production	Excess cytosolic dopamine
VMAT2 sequestration [6]	Prevents oxidation	Quinones
ALDH detoxification [20]	Removes DOPAL	Protein adducts
Phase II conjugation [6]	Metabolite elimination	Reactive metabolite accumulation
Antioxidant buffering [10]	Neutralizes ROS	Oxidative stress
Neuromelanin [9]	Metal sequestration	Iron-mediated toxicity

ALDH, aldehyde dehydrogenase; BH_4_, tetrahydrobiopterin; DOPAL, 3,4-dihydroxyphenylacetaldehyde; ROS, reactive oxygen species; TH, tyrosine hydroxylase; VMAT2, vesicular monoamine transporter 2.

**Table 3:** Reactive intermediates generated during dopamine metabolism.

Intermediate	Formation	Chemical Properties	Molecular Targets	Consequences
Dopamine [6]	TH/AADC	Catechol	Protein thiols	Oxidation
Semiquinone [76]	Autooxidation	Radical	Lipids	ROS
Quinone [76]	Oxidation	Electrophile	Cysteines	Protein dysfunction
DOPAL [20]	MAO	Aldehyde	Lysines	α-synuclein aggregation
H_2_O_2_ [6]	MAO	ROS precursor	Fe^2+^	Hydroxyl radicals
Hydroxyl radical [9]	Fenton	Highly reactive	DNA, proteins	Cell death

AADC, aromatic L-amino acid decarboxylase; DNA, deoxyribonucleic acid; DOPAL, 3,4-dihydroxyphenylacetaldehyde; Fe^2+^, ferrous iron; H_2_O_2_, hydrogen peroxide; MAO, monoamine oxidase; ROS, reactive oxygen species; TH, tyrosine hydroxylase.

**Table 4:** Key knowledge gaps and future research priorities.

Knowledge Gap	Key Question for Future Studies
Higher-order organisation of dopamine metabolism	Do stable functional microdomains or substrate-channelling mechanisms exist in native human dopaminergic neurons, and do they materially influence metabolic flux?
Cofactor dynamics	How do BH_4_, FAD, SAM, and related cofactors vary spatially and temporally within dopaminergic neurons during physiological activity and disease?
Reactive dopamine intermediates	What are the in vivo concentrations, compartmental distribution, and toxicity thresholds of DOPAL and dopamine-derived quinones?
Human relevance of experimental mechanisms	Which mechanisms identified in biochemical, cellular, and animal models are quantitatively relevant to human PD?
Cellular heterogeneity and intercellular coupling	How do neurons, astrocytes, microglia, and other cell types cooperate in dopamine metabolism, detoxification, and redox buffering?
Mechanism-based biomarkers	Can early alterations in dopamine compartmentalization, cofactor status, aldehyde burden, or redox chemistry be measured reliably in patients?
Therapeutic validation	Which mechanistically plausible targets can be modulated safely in humans, and does target engagement translate into altered disease progression?

## Data Availability

No new data were created or analysed in this study. Data sharing is not applicable to this article. All graphs presented in the figures are schematic or representative illustrations and were not generated from original experimental or clinical data.

## Abbreviations

The following abbreviations are used in this manuscript:

AADC	Aromatic L-amino acid decarboxylase
ALDH	Aldehyde dehydrogenase
ALDH1A1	Aldehyde dehydrogenase 1 family member A1
ALDH2	Aldehyde dehydrogenase 2
BH_4_	Tetrahydrobiopterin
COMT	Catechol-O-methyltransferase
DAT	Dopamine transporter
DOPAC	3,4-Dihydroxyphenylacetic acid
DOPAL	3,4-Dihydroxyphenylacetaldehyde
FAD	Flavin adenine dinucleotide
GCH1	GTP cyclohydrolase 1 gene
GTPCH	GTP cyclohydrolase I
HVA	Homovanillic acid
iNOS	Inducible nitric oxide synthase
iPSC	Induced pluripotent stem cell
L-DOPA	L-3,4-Dihydroxyphenylalanine (levodopa)
MAO	Monoamine oxidase
NM	Neuromelanin
PD	Parkinson’s disease
PLP	Pyridoxal 5′-phosphate
RNS	Reactive nitrogen species
ROS	Reactive oxygen species
SAM	S-Adenosyl-L-methionine
SULT	Sulfotransferase
TH	Tyrosine hydroxylase
UGT	UDP-glucuronosyltransferase
VMAT2	Vesicular monoamine transporter 2
